# ZNF652 exerts a tumor suppressor role in lung cancer by transcriptionally downregulating cyclin D3

**DOI:** 10.1038/s41419-024-07197-1

**Published:** 2024-11-05

**Authors:** Chunfeng Xie, Xu Zhou, Jinyi Wu, Weiyi Chen, Dongxue Ren, Caiyun Zhong, Zili Meng, Ye Shi, Jianyun Zhu

**Affiliations:** 1 Department of Nutrition and Food Safety, School of Public Health, Nanjing Medical University, Nanjing, China; 2 Department of Respiratory and Critical Care Medicine, The Affiliated Huai’an No.1 People’s Hospital of Nanjing Medical University, Huai’an, Jiangsu P. R. China; 3 Department of Thoracic Surgery, Nanjing Chest Hospital, Affiliated Nanjing Brain Hospital, Nanjing Medical University, Nanjing, China; 4 Department of Laboratory, The Affiliated Suzhou Hospital of Nanjing Medical University, Suzhou Municipal Hospital, Gusu School, Nanjing Medical University, Suzhou, China

**Keywords:** Lung cancer, Transcription

## Abstract

Dysfunction of zinc finger protein 652 (ZNF652) is associated with various malignant tumors. However, the role of ZNF652 in lung cancer (LC) is poorly understood. Here, we identified that ZNF652 was downregulated in human LC tissues and cell lines. Low ZNF652 expression was associated with poor survival in LC patients. Overexpression of ZNF652 inhibited cell viability, proliferation, migration, and invasion of LC cells, whereas ZNF652 knockdown promoted these malignant phenotypes. Using RNA-seq analysis revealed that ZNF652 overexpression resulted in obvious alterations of various biological processes, especially cell cycle and cellular senescence. Subsequently, we confirmed that ZNF652 overexpression arrested the cell cycle at the G1 phase, increased ROS-mediated DNA damage, induced LC cell senescence, and enhanced cisplatin-induced apoptosis in LC cells. Mechanistically, ZNF652 directly bound to the promoter of cyclin D3 (CCND3), inhibited its transcription, thereby arresting the cell cycle at the G1 phase. Ectopic expression of cyclin D3 rescued the decreased cell viability and cell cycle arrest induced by ZNF652. In vivo studies further showed that ZNF652 overexpression suppressed the tumorigenic potential of LC. Collectively, our findings reveal that ZNF652 exerts a tumor suppressor role in lung cancer by inducing cell cycle arrest and cellular senescence via transcriptionally downregulating cyclin D3. Thus, ZNF652 may be a prognostic predictive factor for LC patients.

## Introduction

Lung cancer (LC) has become the primary reason for cancer-related death worldwide, killing about 1.8 million people in 2022 [1]. It is divided into two main types: small cell lung cancer (SCLC, 15% of cases) and non-small cell lung cancer (NSCLC, 85% of cases). Among NSCLC, the most common primary subtype is lung adenocarcinoma (LUAD), followed by lung squamous cell carcinoma (LUSC) and large cell carcinoma (LCC) [2]. In the past decades, the emergence of targeted and immune-based therapies has largely changed the strategy of NSCLC treatment [3]. Unfortunately, the overall NSCLC survival rate remains very low. Nowadays, with the development of technology, rapid progress has been made in the identification of differentially expressed genes to further understand molecular mechanisms and identify key factors that drive LC complex pathogenesis.

As the largest group of transcription factors, the C2H2 zinc finger (ZNF) proteins have been found to play critical roles in numerous important biological processes, such as cell differentiation, apoptosis, and stemness [4, 5]. Growing evidence indicates that aberrant expression of C2H2 ZNF proteins significantly correlates with tumorigenesis in various types of cancers. ZNF652, a member of the C2H2 ZNF protein family, has been identified to exert tumor suppressive function on oncogenesis, including breast cancer [6], gastric cancer [7], and osteosarcoma [8]. Kumar et al revealed that genes with ZNF652 binding sites function in various cellular pathways, and go together with carcinogenesis [9]. Liu Y et al reported that ZNF652 overexpression inhibited PD-L1 transcription in triple-negative breast cancer [10]. A recent study carried out by Xu J et al showed that a high level of ZNF652 was positive with a good prognosis in 103 primary LUAD samples with paired non-cancerous adjacent tissues (NATs) from treatment-naive Chinese patients (GSE140343) [11]. However, the biological function of ZNF652 in LC is unknown. Therefore, the expression, function, and potential signaling mechanism of the ZNF652 deserves further exploration.

The cell cycle of normal cells is precisely regulated by various checkpoints, while the cell cycle of cancer is deregulated, leading to uncontrolled cell proliferation. Overexpression or gain-of-function mutations of cyclins and cyclin-dependent kinases (CDKs) are extremely common in the aberrant cell cycle progression of cancers [12]. As the important regulators of the G1/S-phase cell cycle transition, D-type cyclins (cyclin Ds) and their related CDKs control cancer cells through various aspects, including proliferation, migration, senescence, and apoptosis [13, 14]. It is important to note that of the D-cyclin family, cyclin D3 is highly dysregulated in a variety of cancers [15, 16], including LC [17, 18]. Furthermore, cyclin D3 expression has been reported to be associated with prognosis and drug resistance in LC [17, 19]. Previous studies have shown that targeting cyclin D3 could induce cell cycle arrest in various tumor types [20–23]. Thus, cyclin D3 could be an attractive therapeutic target in LC.

In this study, we explored the functions of ZNF652 in the LC progression. ZNF652 was downregulated in LC tissues and cell lines. Moreover, ZNF652 level was associated with patient survival. We further identified ZNF652 as a tumor suppressor and elucidated its mechanisms in inhibiting the cell and tumor growth of LC.

## Materials and methods

### Bioinformatics analysis

To analyze the ZNF652 expression in LC, gene expression profiling datasets were obtained from the Gene Expression Omnibus (GEO) database (GSE11969 and GSE43458). The Human Protein Atlas (HPA) (http://www.proteinatlas.org/) and the Kaplan-Meier plotter (http://kmplot.com) were used to validate the survival curve analysis of LC patients with ZNF652 expression. Additionally, the expression of ZNF652 in different cancer stages (stage I-IV) of LUAD and LUSC were downloaded from Gene Set Cancer Analysis (GSCA) (http://bioinfo.life.hust.edu.cn/GSCA/#/).

### Clinical samples

A total of 30-paired surgical LC tumor tissues and paired normal adjacent tissues(NATs) were obtained from Huai’an First People’s Hospital affiliated with Nanjing Medical University. The clinical pathological characteristics of the tissues are shown in Table S1. All the tissues were histologically examined by experienced pathologists according to World Health Organization (WHO) criteria. The study was approved by the Ethics Committee of Nanjing Medical University with ethical clearance application number (2016–318), and all patients signed informed consent.

### RNA extraction and Quantitative RT-PCR (RT-PCR)

The procedures for RNA isolation, cDNA preparation, and RT-PCR were performed as described previously [24]. The primers are listed in Table S2. Each sample was run in triplicate.

### Western blotting (WB) analysis

The protocol was performed as described previously [24]. The primary antibody against ZNF652 (#TA329381) was purchased from Origene Technologies (Rockville, MD, USA). Primary antibodies against E-cadherin (#20874-1-AP), Vimentin (#22031-1-AP), p53 (#10442-1-AP), p21 (#10355-1-AP), cyclin A2 (#18202-1-AP), cyclin E1 (#11554-1-AP), CDK2 (#10122-1-AP), Cdc25A (#55031-1-AP), E2F1 (#66515-1-Ig), cyclin D1 (#26939-1-AP), CDK4 (#11026-1-AP), Skp2 (#15010-1-AP), cleaved caspase 3 (#19677-1-AP) and cyclin D3 (#26755-1-AP) were purchased from Proteintech (Wuhan, China). The primary antibody against phospho-γH2AX (Ser139) (#2577) was purchased from Cell Signaling Technology (Danvers, MA, USA). The primary antibody against GAPDH (#MB66349) was purchased from Bioworld (Nanjing, China).

### Cell lines and drug treatments

Normal human bronchial epithelial (NHBE) cells were purchased from the Xiang Ya Central Experiment Laboratory (Changsha, China). Human LC cell lines A549, H1299, and H460 were obtained from Chinese Academy of Typical Culture Collection Cell Bank (Shanghai, China). The cells were routinely cultured at 37 °C in RPMI-1640 (Gibco, Carlsbad, CA, USA), supplemented with 10% fetal bovine serum, 100 IU/mL penicillin, and 100 μg/mL streptomycin. To analyze the effects of cisplatin (CDDP, Sigma Aldrich, St. Louis, MO, USA) and CDK4/6 inhibitor (Palbociclib, Selleck Chemicals, Houston, TX, USA) on LC, the cells were treated with 2 μg/mL CDDP, or 2 μM Palbociclib for 48 h, and subjected to the subsequent experiments.

### Lentiviral vectors and construction of stable cell lines

Lentivirus encoding ZNF652 (HBLV-h-ZNF652-3xflag-LUC-PURO), control vector lentivirus (pHBLV-CMV-MCS-3flag-EF1-fLUC-T2A-PURO), ZNF652-shRNA (shZNF652) and control shRNA (shCtrl) were obtained from Hanbio Biotechnology (Hefei, China). The shRNA sequence was as follows: control shRNA is 5’-TTCTCCGAACGTGTCACGTAA-3’, for shZNF652-1 is 5’-GTAGAGAAAGTCAGCGTTA-3’, for shZNF652-2 is 5’-GAGAAGTGCTTTCGGGTGA-3’, for shZNF652-3 is 5’- GCACATGAACGTTACTCATAG-3’.

For generation of ZNF652 stably expressing or knockdown cell lines, A549 and H460 cells were transfected with ZNF652 lentivirus, vector control lentivirus, ZNF652 shRNA or control shRNA respectively, according to the product protocol. 24 h post-transfection, cells were selected with 2 μg/mL puromycin (Beytime) for 2 weeks. The stable cell clones were selected and maintained in 0.2 μg/mL puromycin. RT-PCR and WB assays were performed for ZNF652 expression identification.

### Plasmids and transfection

CCND3 overexpression plasmid pcDNA3.1-CCND3 (CCND3) and negative control pcDNA3.1 (NC) were obtained from KeyGEN BioTECH (Nanjing, China). To identify the regulation role of ZNF652 on cyclin D3, the vector control cells and ZNF652-OE A549 and H460 cells were transfected with CCND3 or negative control plasmids, using lipofectamine 3000 (Invitrogen, Carlsbad, CA, USA) in Opti-MEM medium according to the manufacturer’s instructions.

### Cell viability, cell proliferation, cell cycle, and apoptosis assay

The detailed procedures for cell viability, cell proliferation, colony formation, cell cycle distributions, and apoptosis were performed as described previously [24]. The viability of ZNF652-OE and shZNF652 LC cells was assessed by cell counting kit-8 (CCK-8) assay (Dojindo, Kumamoto, Japan). The role of ZNF652 on cell proliferation were determined by 5-ethynyl-2’-deoxyuridine (EdU) cell proliferation kit (Beytime, Shanghai, China) and a colony formation assay via crystal violet staining (Beyotime). The cell cycle and apoptosis were measured by flow cytometry using a propidium iodide (PI)/RNase staining kit (BD, Biosciences, USA) and Annexin V /PI apoptosis detection assay kit (BD, Biosciences, USA).

### Cell migration and invasion assays

For cell migration assay, ZNF652-OE or shZNF652 LC cells (3 × 10^4^ cells/well) were seeded with a total volume of 200 μL serum-free medium in the upper chambers (24-well migration chambers, 8.0 μm pore membrane, Corning, New York, USA). The lower chambers were filled with 800 μL of the medium with 10% FBS. After 48 h incubation, the remaining non-migrated cells in the upper chambers were removed, and migrated cells on the undersurface of the chambers were stained with crystal violet. The same procedure was followed for the invasion assay, except for precoating with Matrigel (Corning) for the upper chamber. The number of migrated and invaded cells was photographed under an inverted microscope (Nikon, Tokyo, Japan) and measured by Image J software version 8.0.

### Flow cytometry analysis of reactive oxygen species (ROS)

The vector control or ZNF652-OE LC cells were cultured into 6-well plates at a density of 1 × 10^5^ cells/well. After incubation with 2’, 7’-dichlorodihydrofluorescein diacetate (5 mM, DCF-DA, Sigma–Aldrich) in the dark at 37 °C for 20 min, the DCF fluorescence intensity was detected using flow cytometry.

### Senescence-associated β-galactosidase staining

The effects of ZNF652 on cell senescence were detected using the senescence β-galactosidase (SA-β-gal) staining kit (Beyotime) as described previously [24]. The vector or ZNF652 overexpressed cells (1 × 10^5^ cells/well) were plated onto 6-well plates. The cells were incubated with SA-β-gal staining solution in the dark at 37 °C overnight. The images were obtained under an inverted microscope (Nikon).

### RNA sequencing and gene set enrichment analysis (GSEA)

ZNF652-OE and vector control A549 cells were treated with TRIzol Reagent. Three independent samples of each group were collected and then were sent to LC-Bio Technology CO., Ltd., Hangzhou, China. The quantity and purity of total RNA were analyzed by NanoDrop ND-1000 and Bioanalyzer 2100, and high-quality RNA samples with RNA integrity number > 7.0 were used to construct a sequencing library. The sequencing library was used for transcriptome sequencing which was performed by Illumina HiSeq 4000 in LC-Bio Hangzhou, China and the sequencing mode was PE150. Differentially expressed genes (DEGs) were selected using the DEseq^2^ with log_2_ (fold change) ≥ 1 or log_2_ (fold change) ≤ −1 and with a statistical significance of *p* < 0.05. An enrichment analysis of Gene Ontology (GO), Kyoto Encyclopedia of Genes and Genomes (KEGG), and Gene Set Enrichment Analysis (GSEA) was performed to gain further insights to the functional analysis of DEGs.

### Immunofluorescence (IF) staining

The LC cells were seeded in 24-well plates and were fixed with 4% paraformaldehyde, permeabilized with 0.25% Triton X-100, and blocked with 5% bovine serum albumin (BSA). The cells were then incubated with primary antibody against E-cadherin (1:400 dilution, #20874-1-AP, Proteintech), Vimentin (1:400 dilution, #22031-1-AP, Proteintech) and phospho-γH2AX (1:400 dilution, # 2577, Cell Signaling Technology) at 4 °C overnight. After incubation with secondary antibody for 45 min, the nuclei were then stained with DAPI for 10 min. The fluorescence images were observed using a fluorescence microscope (Nikon).

### Chromatin immunoprecipitation (ChIP) assay

A549 cells were collected and cross-linked in 1% formaldehyde for 10 min. The fixed cells were then sonicated to obtain chromatin fragments. Subsequently, the samples were incubated with ZNF652 antibody (Origene Technologies) and anti-IgG (Cell Signaling Technology). After incubation, protein A/G beads were applied to capture the samples for 2 h according to the manufacturer’s instruction (Cell Signaling Technology). Purified DNA was used for RT-PCR analysis. The primers encompassing the ZNF652 binding sites in different regions of the cyclin D3 promoter were designed as follows: CCND3-BS1 F, AGAGTGAAGTGTTCTGGGGC, CCND3-BS1 R, AGATGATCTCCTGGTATGCTCT; CCND3-BS2 F, GTCACAGTGCCTGACGTGTA, CCND3-BS2 R, TCCTGACCTGAGGTGATCCA.

### Dual-luciferase reporter assay

The CCND3 promoter region (−2000 to +50) with ZNF652 binding site or mutated site was cloned into psiCHECK plasmid by Genomeditech (shanghai, China), termed CCND3-wt and CCND3-mut. The vector control and ZNF652-OE A549 cells (1 × 10^5^ cells/well) were seeded in a 24-well plate 24 h before transfection. Cells were then transfected with CCND3-wt or CCND3-mut reporter gene plasmids for 48 h. The luciferase activities were evaluated by a dual luciferase kit (Promega, Madison, WI, USA) according to the manufacturer’s instructions. Data were expressed as relative luciferase activity (Firefly luciferase activity/Renilla luciferase activity). Each experiment was performed in triplicate.

### Animal studies

The ZNF652-OE and vector control A549 cells were harvested, counted, and resuspended in PBS. The cells (5 × 10^6^ cells/100 μl) were then transplanted into the right axilla of 6-week-old 16–18 g male BALB/c nude mice (8 mice/group, Animal Research Center, Nanjing Medical University). The tumors were measured every 5 days using a caliper and tumor volume was calculated using a formula: [length × width^2^] / 2, where length and wide are the major and minor diameters. The experiment was terminated when the tumors reached an appropriate size. Finally, tumor tissues were collected and frozen for WB analysis or embedded for histochemical and immunohistochemistry evaluation. The animal experiments were approved by the Animal Care and Welfare Committee of Nanjing Medical University (IACUC-1907001).

### Histochemistry and immunohistochemistry staining

The surgical specimens and mice tumor tissues were fixed, dehydrated, and embedded in paraffin for slicing into 5 μm slides. Afterward, the slides were used for hematoxylin-eosin (HE) or immunohistochemical (IHC) staining. The antibodies used for IHC were rabbit anti-ZNF652 (1:200 dilution, #TA329381, Origene), rabbit anti-PCNA (1:200 dilution, #10205-2-AP, Proteintech), rabbit anti-cleaved caspase 3 (1:200 dilution, #19677-1-AP, Proteintech), rabbit anti-cyclin D3 (1:200 dilution, #26755-1-AP, Proteintech), rabbit anti-cyclin A2 (1:200 dilution, #18202-1-AP, Proteintech), and rabbit anti-cyclin E1 (1:200 dilution, #66515-1-Ig, Proteintech). Finally, representative images were taken under an optical microscope (Nikon).

### Statistical analysis

Each experiment was repeated at least three times. All data were represented as mean ± standard deviation (SD). The mRNA expression between paired lung cancer patient tumor tissues was determined by paired two-tailed student’s *t*-test. Statistical analysis between the two groups was compared using an unpaired two-tailed Student’s t-test. Statistics analysis among multiple groups was compared using one-way analysis of variance (ANOVA). A *P* value < 0.05 was considered a significant difference.

## Results

### The expression of ZNF652 was downregulated in LC

**Fig. 1:**
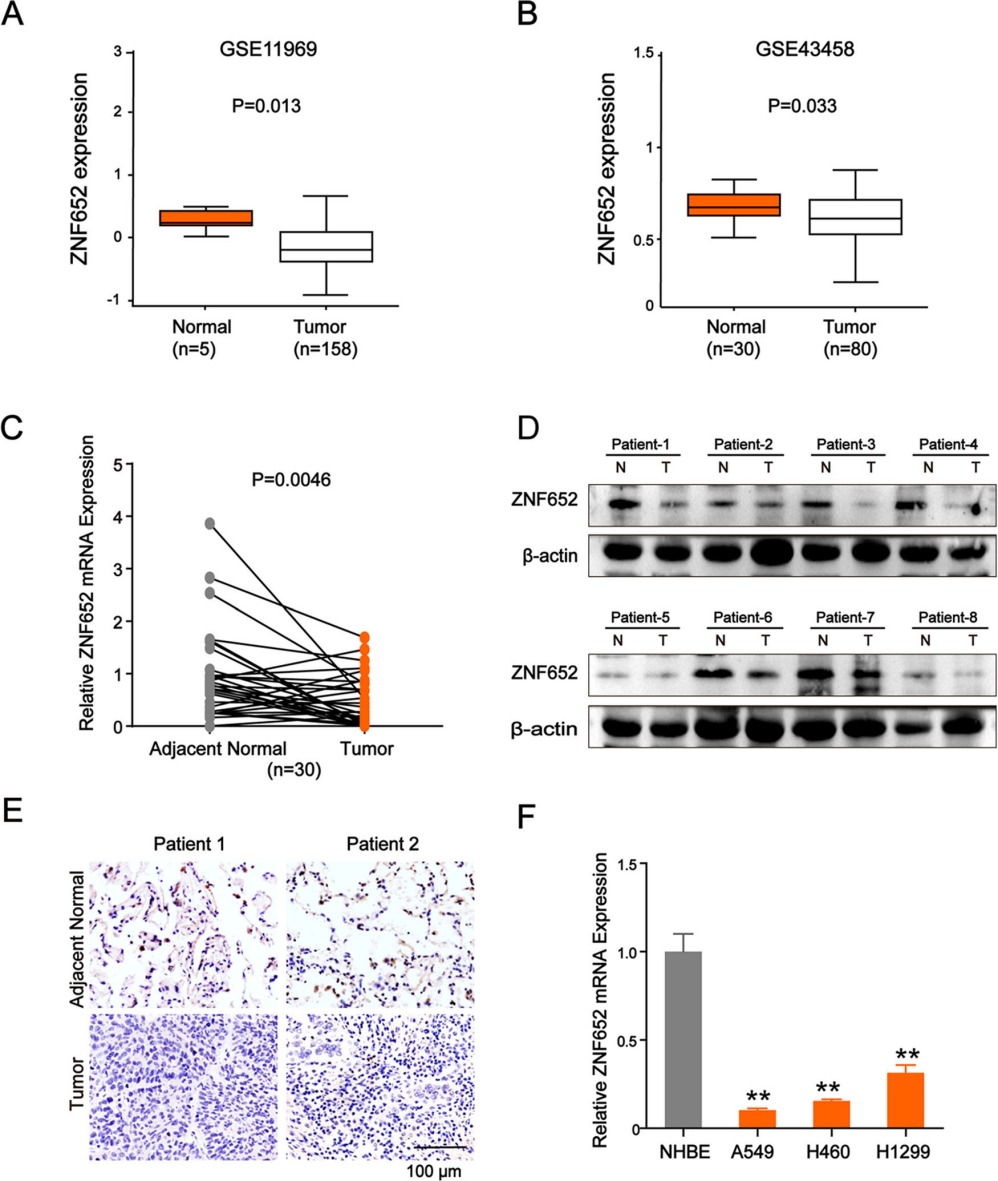
ZNF652 expression was downregulated in LC. **A**, **B**. The expression of ZNF652 in LC based on GEO datasets (GSE11969 and GSE43458). **C** The mRNA expression of ZNF652 in LC tissues and paired adjacent normal lung tissues (*n* = 30). GAPDH was served as an internal reference. *p* = 0.0046, paired *t*-test. **D** The protein expression of ZNF652 in LC tissues and paired adjacent normal lung tissues of eight patients. β-actin served as an internal reference. **E** Representative IHC images of ZNF652 in LC tissues and paired adjacent normal lung tissues. Scale bars, 100 μm. **F** The mRNA expression of ZNF652 in normal human bronchial epithelial (NHBE) cells and LC cells (A549, H460, and H1299) was shown. The data are presented as mean ± SD (*n* = 3). **p < 0.01 *vs*. NHBE group.

To investigate the characteristics of ZNF652 in LC, we first performed an online analysis using the UCSC XenaShiny based on the cancer genomic atlas pan-cancer data and GEO datasets (GSE11969 and GSE43458). Results revealed that ZNF652 was decreased in LC tissues compared to normal lung tissues, including LUAD and LUSC (Fig. S1 and Fig. 1A, B). To validate this observation, we collected 30 surgical lung cancer specimens in clinical and examined the ZNF652 expression in lung cancer tissues and normal adjacent tissues. RT-PCR and WB analysis showed that when compared to the normal adjacent tissues, both the mRNA and protein levels of ZNF652 were significantly lower in LC tissues (Fig. 1C, D). Furthermore, the IHC staining assay demonstrated a decreased expression of ZNF652 in LC tissues compared to paired normal tissues (Fig. 1E).

Besides, to identify the ZNF652 expression in established cell line, we detected the mRNA level of ZNF652 in normal human bronchial epithelial (NHBE) cells and LC cell lines (A549, H460, and H1299). The data showed that ZNF652 expression was lower in LC cells than that in NHBE cells (Fig. 1F). These results suggested that ZNF652 was downregulated in LC.

### Expression of ZNF652 was associated with clinical outcomes of LC patients

**Fig. 2:**
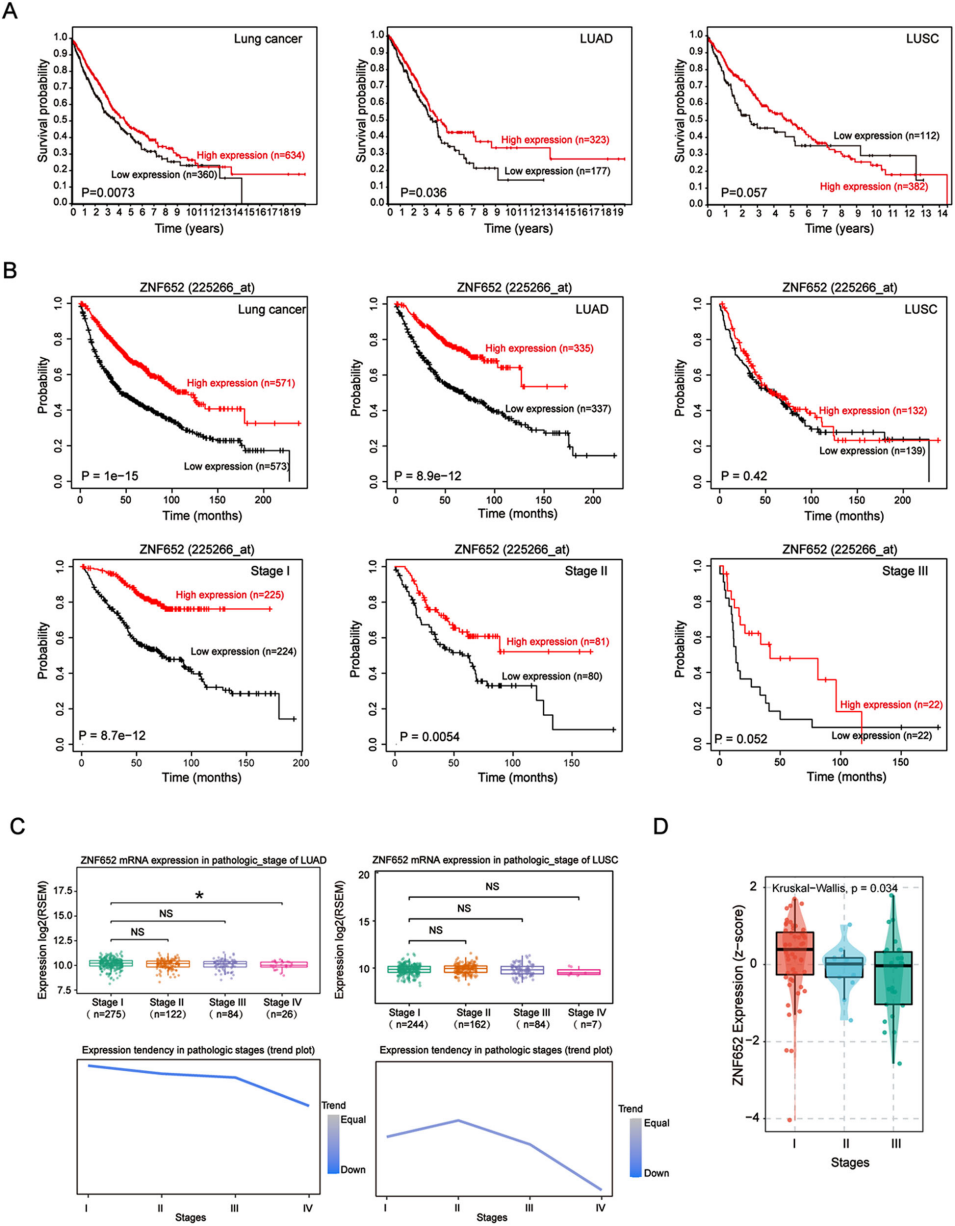
The expression of ZNF652 was correlated with clinical outcomes of LC patients. **A** OS analysis for LC patients with high or low levels of ZNF652 using the HPA dataset database. **B** Kaplan–Meier survival analysis of OS for LC patients with high or low levels of ZNF652 expression. **C** GSCA analysis of ZNF652 expression level and trend in different stages in LUAD and LUSC. **D** Expression level of ZNF652 in different stages in the GEO database (GSE11969).

Furthermore, to assess the clinical significance of ZNF652 in LC, we evaluated the correlation between ZNF652 level and the clinical outcomes of LC patients. We queried the Human Protein Altas (HPA) dataset (https://www.proteinaltas.org), it showed that reduced ZNF652 level was associated with poor overall survival (OS) in LC, particularly in LUAD (Fig. 2A). In parallel, Kapan-Meier survival analysis (http://kmplot.com) revealed that the patients with higher level of ZNF652 had longer OS than those with lower level of ZNF652, except LUSC (Fig. 2B). Subgroup analysis showed that patients with high ZNF652 expression exhibited better OS rates compared with those with low ZNF652 expression in stage I and II (Fig. 2B). The survival probability analysis indicated that LC patients with greater expression of ZNF652 held better outcomes than those with lower ZNF652 expression.

To further explore the role of ZNF652 in LC progression, the trends of ZNF652 expression were analyzed in different pathological stages in LC and GEO database (GSE11969). Gene Set Cancer Analysis (GSCA) (http://bioinfo.life.hust.edu.cn/GSCA/#/) showed that ZNF652 expression of Stage IV LUAD tissues was remarkably lower than that of Stage I. Significantly, the expression of ZNF652 exhibited a decreasing tendency following the progression of the pathological stages (Fig. 2C, D). Next, we performed a comprehensive analysis of genetic alterations of ZNF652 in lung cancer using cBioPortal for Cancer Genomics (http://www.cbioportal.org). As shown in Fig. S2, ZNF652 was frequently mutated in LC. These findings indicated that ZNF652 functioned as a tumor suppressor in LC.

### Ectopic expression of ZNF652 inhibited LC cell proliferation in vitro

**Fig. 3:**
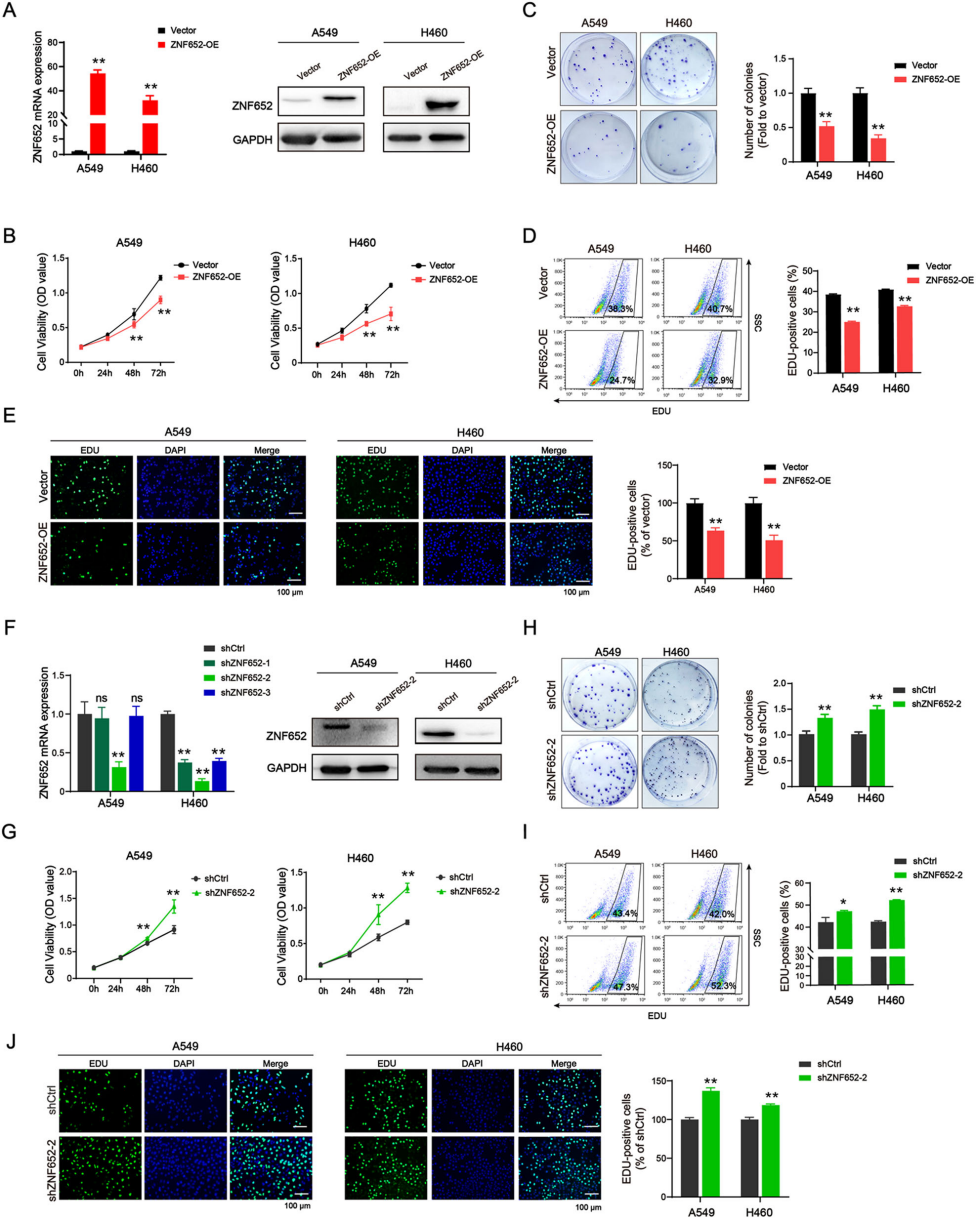
ZNF652 overexpression suppressed cell viability, colony formation, and cell proliferation in vitro. **A** A549 and H460 cells transfected with empty control vector (Vector) or ZNF652 overexpression lentivirus (ZNF652-OE) were harvested. Expression of ZNF652 in ZNF652-OE and vector control cells were examined using RT-PCR and WB analysis. **B** Cell viability was measured at 0, 24, 48, and 72 h in vector control and ZNF652-OE LC cells. **C** Colony formation ability of vector control and ZNF652-OE LC cells were detected. Histograms of relative colony formation rates from three independent experiments. **D**, **E** EdU assay was performed to examine the proliferation of vector control and ZNF652-OE LC cells. Flow cytometry analysis of EdU staining positive cells (**D**). Images of EdU-stained cells and histograms of the percentage of EdU-stained cells were shown (**E**). Scale bars, 100 μm. **F** A549 and H460 cells transfected with control shRNA (shCtrl) or three different shZNF652 were detected for mRNA expression using RT-PCR. ZNF652 protein expression was detected in LC cells transfected with shZNF652-2 and shCtrl using WB analysis. **G** Cell viability was measured at 0, 24, 48, and 72 h in shCtrl and shZNF652-2 LC cells. **H** Colony formation ability of shCtrl and shZNF652-2 LC cells was detected. Histograms of relative colony formation rates from three independent experiments. **I**, **J** EdU assay was performed to examine the proliferation of shCtrl and shZNF652-2 LC cells. Flow cytometry analysis of EdU staining positive cells (**I**). Images of EdU-stained cells and histograms of the percentage of EdU-stained cells were shown (**J**). The data are presented as mean ± SD (*n* = 3). ns, no significant, *p < 0.05, **p < 0.01 *vs*. Vector or shCtrl group.

Given the frequent downregulation of ZNF652 in LC tissues and LC cell lines, we speculate that ZNF652 may function as a tumor suppressor in lung tumorigenesis. Therefore, in vitro studies were performed to determine the function of ZNF652 in LC. First, the LC cell lines A549 and H460 with stable overexpression or knockdown of ZNF652 were established. As shown in Fig. 3A, compared to the vector control cells, ZNF652 mRNA and protein levels were markedly increased in ZNF652-OE A549 and H460 cells. Conversely, the expression of ZNF652 in shZNF652-2 was significantly lower than in the control shRNA (shCtrl) cells (Fig. 3F). These results identified ZNF652 expression status.

Next, MTT assays showed that overexpression of ZNF652 potently decreased cell viability of A549 and H460 at 48 h and 72 h (Fig. 3B). Moreover, the colony formation and the percentage of Edu-positive cells in ZNF652 overexpressed LC cells were remarkably reduced, compared to vector control cells (Fig. 3C–E). In contrary, knockdown of ZNF652 significantly increased cell viability, promoted the ability of colony formation, and enhanced cell proliferation in A549 and H460 cells (Fig. 3G–J). The above results illustrated that ZNF652 exerted anti-tumorigenic activity in vitro.

### Ectopic expression of ZNF652 suppressed LC cell migration/invasion in vitro

**Fig. 4:**
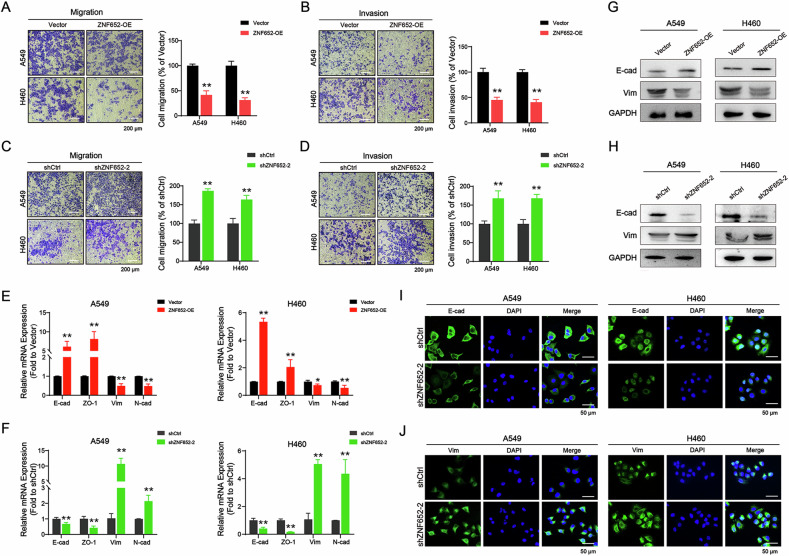
ZNF652 overexpression repressed LC cell migration and invasion through reversing EMT in vitro. **A**, **B** Effects of ZNF652 overexpression on the cellular migration (**A**) and invasion (**B**) ability were measured by transwell assays with or without Matrigel. Representative images were photographed following fixation and staining. Scale bars, 200 μm. **C**, **D** Effects of ZNF652 knockdown on the cellular migration (**C**) and invasion (**D**) ability were measured by transwell assays with or without Matrigel. Representative images were photographed following fixation and staining. Scale bars, 200 μm. **E**, **F** Expression of EMT-related proteins E-cad, ZO-1, Vim, and N-cad was detected in ZNF652 upregulated or downregulated A549 and H460 cells using RT-PCR. **G, H**. Expression of E-cad and Vim in ZNF652 upregulated or downregulated LC cells was detected using WB assay. **I**, **J** Expression of E-cad and Vim in ZNF652 downregulated LC cells was detected using IF staining assay. Representative images of E-cad and Vim are shown. Green, E-cad or Vim; blue, DAPI. Scale bars, 50 μm. The data are presented as mean ± SD (*n* = 3). *p < 0.05, **p < 0.01 *vs*. Vector or shCtrl group.

Neilsen et al reported that loss of ZNF652 in primary breast tumors correlated with increased invasion and metastasis [25]. Subsequently, to determine the role of ZNF652 in the metastasis of LC, Transwell assay was used to evaluate the cell motility. The results showed that cellular migration and invasion ability were reduced in ZNF652-OE A549 and H460 cells (Fig. 4A, B), while ZNF652 knockdown markedly enhanced the metastatic capacity of LC cell lines (Fig. 4C, D). Moreover, RT-PCR analysis showed that the upregulation of ZNF652 upregulated the expression of E-cadherin (E-cad) and ZO-1 and downregulated the levels of Vimentin (Vim) and N-cadherin (N-cad) (Fig. 4E). WB analysis further showed a similar change of E-cad and Vim expression in ZNF652-OE A549 and H460 cells (Fig. 4G). On the contrary, shZNF652 LC cells have the opposite effects (Fig. 4F, H, I, J). These results suggested that ectopic expression of ZNF652 appeared to inhibit the LC cell metastasis by reversal of the EMT process.

### Transcriptome analysis revealed that ZNF652 was closely correlated with functional changes in LC cell cycle

**Fig. 5:**
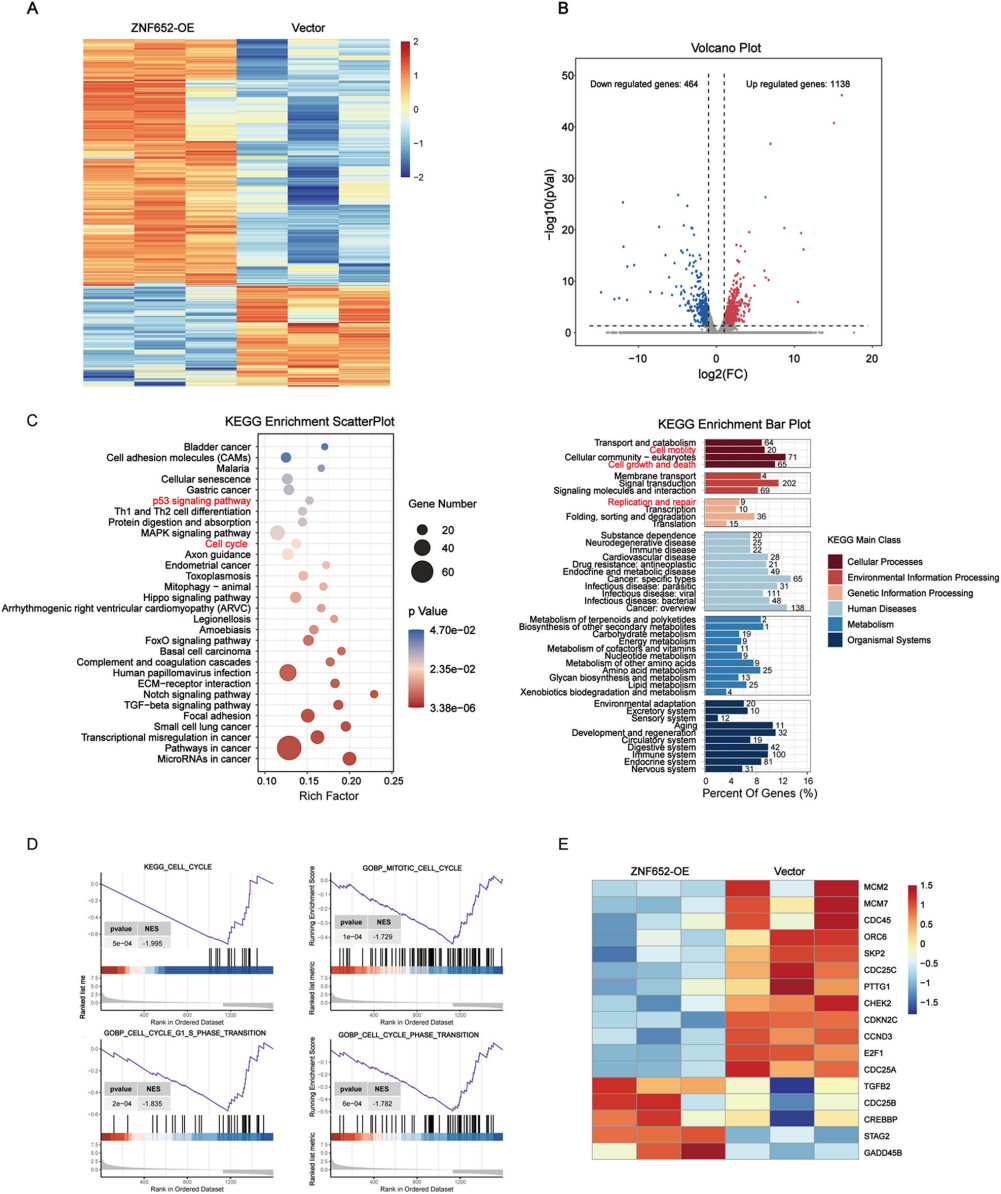
RNA-seq analysis revealed features of the cell cycle in ZNF652 overexpressed LC cells. **A** Heatmap showing the gene expression changes after ZNF652 overexpression in A549 cells. **B** Volcano plot showing differentially expressed genes (DEGs). Red plots, the gene significantly upregulated in ZNF652-OE A549 cells compared with vector control cells. Blue plots, genes significantly downregulated. **C** KEGG pathway analysis of the DEGs, the top 30 enriched pathways, and KEGG main class. **D** GSEA analysis was performed using A549 cells with ZNF652 overexpression and vector control cells. **E** Heatmap showing the ZNF652-altered genes involved in the cell cycle.

To better explore the potential mechanism whereby ZNF652 overexpression might inhibit lung cancer cell growth and metastasis, transcriptional profiling was performed on ZNF652-OE and vector control A549 cells. As shown in Fig. 5A, ZNF652 overexpression markedly altered the transcriptome of A549 cells. We analyzed the difference in gene expression between the two cell groups and found that 1138 genes were upregulated whereas 464 genes were downregulated upon ZNF652 overexpression (Fig. 5B).

To explore which biological pathways are mostly affected by ZNF652, we applied a Kyoto Encyclopedia of Genes and Genomes (KEGG) analysis of the differentially expressed genes (DEGs). We found that ZNF652 overexpression resulted in obvious alterations of various biological processes, such as cell cycle and p53 signaling pathways. Further analysis found that those target genes were enriched in the regulation of cell motility, cell growth and death, replication and repair (Fig. 5C). Next, we evaluated the ZNF652-related pathway using GSEA, the data also showed that the enriched genes are associated with cell cycle pathway, mitotic cell cycle, cell cycle G1-S phase transition and cell cycle phase transition (Fig. 5D). Indeed, by visualizing the changes with volcano plot and heat map, we noted that host of G1-S phase marker genes was down-regulated by ZNF652 overexpression, such as CDC25A, E2F1, CCND3, CDKN2C, CDC25C, SKP2, et al (Fig. 5E). These findings collectively indicated that ZNF652 overexpression induced a decrease of many genes involved in cell cycle, resulting in the alteration of regulation of cell motility, cell growth and death, and replication and repair.

### ZNF652 overexpression arrested LC cells at the G1 phase

**Fig. 6:**
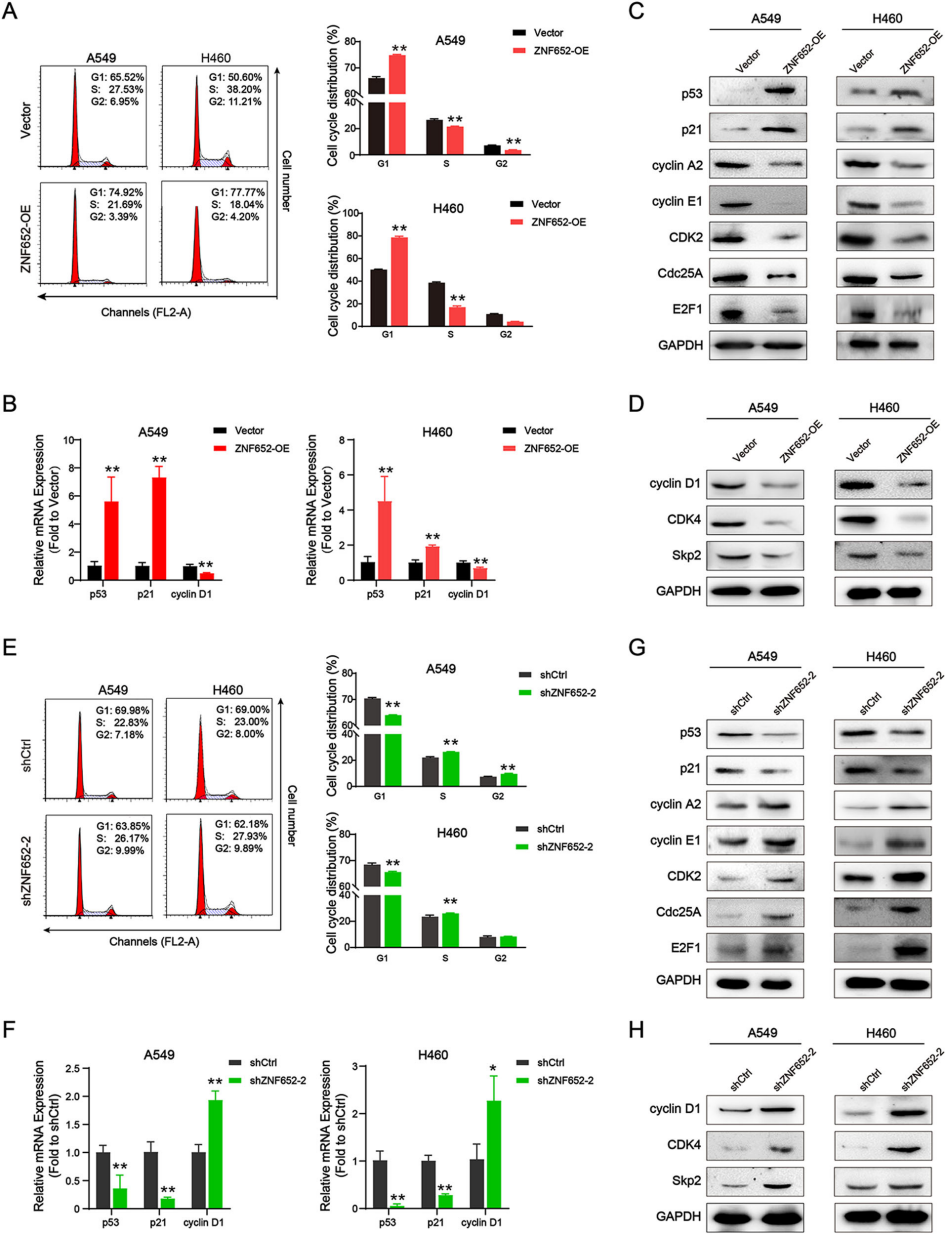
ZNF652 induced LC cell cycle arrest at the G1 phase. **A** Cell cycle distribution measured in vector control and ZNF652-OE A549 and H460 cells using flow cytometry assay. **B** p53, p21, and cyclin D1 mRNA expression were detected in vector control and ZNF652-OE LC cells using RT-PCR assay. **C**, **D** Expression of cell-cycle related protein (p53, p21, cyclin A2, cyclin E1, Cdc25A, CDK2, E2F1, cyclin D1, CDK4, and Skp2) was detected in vector control cells and ZNF652-OE LC cells using WB assay. **E** Cell cycle distribution measured in shCtrl and shZNF652-2 A549 and H460 cells using flow cytometry assay. **F** p53, p21, and cyclin D1 mRNA expression were detected in shCtrl and shZNF652-2 LC cells using RT-PCR assay. **G**, **H**. Expression of cell-cycle related protein (p53, p21, cyclin A2, cyclin E1, Cdc25A, CDK2, E2F1, cyclin D1, CDK4, and Skp2) was detected in shCtrl and shZNF652-2 LC cells using WB assay. The data are presented as mean ± SD (*n* = 3). *p < 0.05, **p < 0.01 *vs*. Vector or shCtrl group.

To validate the above results, we carried out flow cytometry to explore the influence of ZNF652 on the cell cycle distribution of LC cells. We observed that ZNF652 overexpression significantly increased the ratio of G1-phase cells in A549 and H460 cells, while reducing the percentage of S-phase in both LC cell lines (Fig. 6A). To determine how ZNF652 blocks the cell cycle in the G1 phase, RT-PCR and WB analysis were used to detect the expression changes of G1 and S phase-related proteins. First, we found that ZNF652 overexpression increased p53 and p21 mRNA levels, and decreased cyclin D1 mRNA levels (Fig. 6B). Consistently, WB assay further showed that ZNF652 overexpression resulted in G1-S phase-related protein alterations. The expression of p53 and p21 protein were also significantly upregulated in ZNF65-OE A549 and H460 cells, whereas cyclin A2, cyclin E1, CDK2, Cdc25A, E2F1, cyclin D1, CDK4, and Skp2 were remarkably downregulated (Fig. 6C, D). On the contrary, ZNF652 knockdown accelerated cell cycle progress in LC cells (Fig. 6E). The mRNA and protein expression of G1-S phase-related genes showed the opposite results in ZNF652 knockdown LC cells (Fig. 6F–H). These results implied that ectopic expression of ZNF652 arrested LC cells at the G1 phase via modulating G1-S phase regulatory mediators.

### ZNF652 overexpression promoted ROS overproduction, induced DNA damage and cellular senescence, enhanced chemotherapy sensitivity in LC cells

**Fig. 7:**
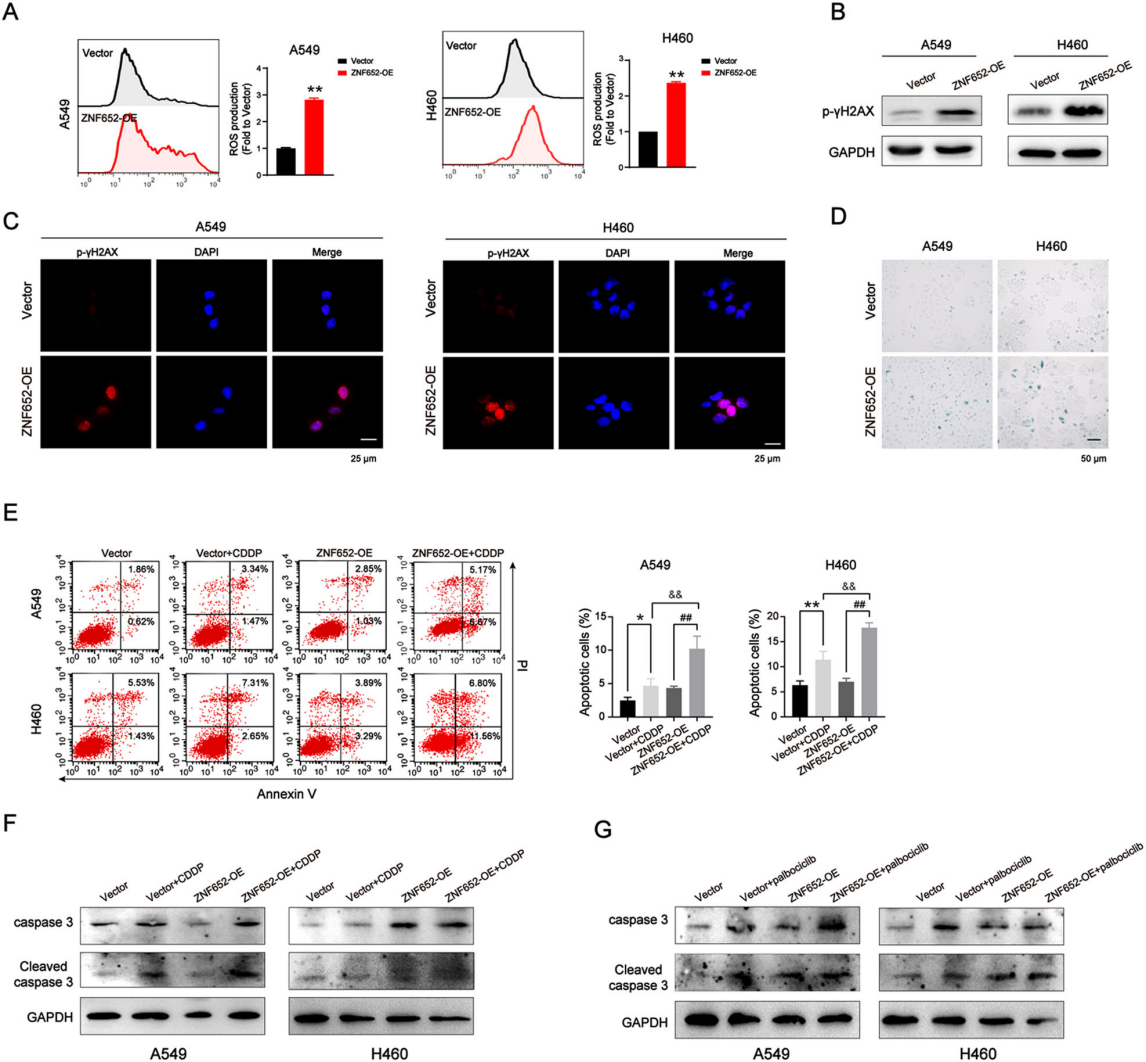
ZNF652 overexpression enhanced the sensitivity of LC cells to CDDP. **A** Flow cytometry analysis of ROS production in vector control and ZNF652-OE A549 and H460 cells. **B** WB analysis of p-γH2AX expression in vector control and ZNF652-OE LC cells. **C** IF staining assay of p-γH2AX expression in vector control and ZNF652-OE LC cells. Red, p-γH2AX, blue, DAPI. Scale bars, 25 μm. **D** Senescence β-galactosidase (SA-β-gal) staining assay of vector control and ZNF652-OE LC cells. Scale bars, 50 μm. **E** Flow cytometry analysis of apoptosis fractions in vector control and ZNF652-OE LC cells following treatment with CDDP. **F** WB analysis of caspase 3 and cleaved caspase 3 in vector control and ZNF652-OE LC cells following treatment with CDDP. **G** WB analysis of caspase 3 and cleaved caspase 3 in vector control and ZNF652-OE LC cells following treatment with palbociclib. The data are presented as mean ± SD (*n* = 3). *p < 0.05, **p < 0.01, *vs*. Vector group; ##*p* < 0.01, *vs*. ZNF652-OE group, &&*p* < 0.01, *vs*. Vector + CDDP group.

Cellular senescence refers to a state of durable cell cycle arrest and plays a crucial role in cancer biology [26]. Due to RNA-seq analysis showing cell senescence in ZNF652-OE LC cells, we further assessed whether ZNF652 overexpression resulted in cellular senescence through various pathways. Firstly, we found that compared with the corresponding control vector cells, ROS production significantly increased in ZNF652-OE A549 and H460 cells (Fig. 7A). Secondly, the protein expression of p-γH2AX, a biomarker of DNA damage, was also elevated in ZNF652-OE A549 and H460 cells (Fig. 7B, C). Specially, the number of SA-β-gal-positive cells increased in ZNF652-OE A549 and H460 cells (Fig. 7D). These results suggested that ZNF652 resulted in cellular senescence in lung cancer via inducing cell cycle arrest, increasing ROS production and impairing DNA replication and repair. Additionally, compared with the cisplatin (CDDP) treated group, ZNF652 overexpression promoted CDDP-induced apoptosis in LC cell lines, accompanied by the increased expression of apoptotic proteins caspase 3 and cleaved caspase 3 (Fig. 7E, F). These findings demonstrated that ZNF652 overexpression made LC cells more sensitive to chemotherapy. Given cyclin D3 and its associated cyclin-dependent kinases CDK4 and CDK6 control the progress of cell cycle, we wondered whether ZNF652 overexpression could synergize with CDK4/6 inhibition. As shown in Fig. 7G, ZNF652 overexpression led to higher levels of apoptotic proteins caspase 3 and cleaved caspase 3 induced by palbociclib (a CDK4/6 inhibitor) in LC cells, indicating that ZNF652 overexpression enhanced LC apoptosis induced by palbociclib. Our above results indicated that ZNF652 overexpression led to the appearance of cellular senescence, enhanced chemotherapy sensitivity, and has a synergistic effect with CDK4/6 inhibitor on LC cell apoptosis.

### ZNF652 transcriptionally downregulated cyclin D3 expression

**Fig. 8:**
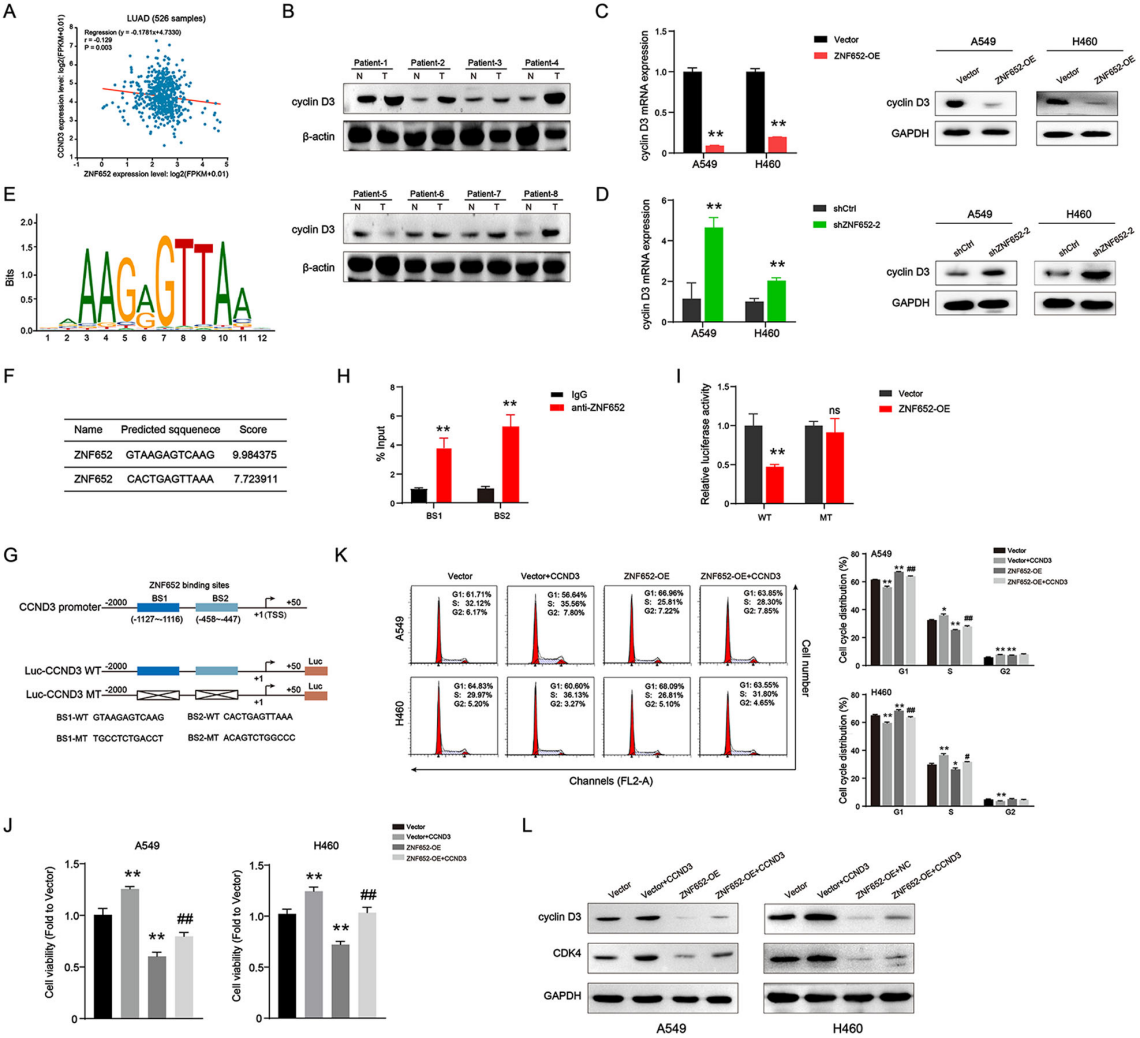
ZNF652 targets cyclin D3 to cause cell cycle arrest at the G1 phase. **A** Correlation between ZNF652 expression and CCND3 level in LUAD. **B** The protein expression of cyclin D3 in LC tissues and paired adjacent normal lung tissues of eight patients. β-actin served as an internal reference. **C**, **D** RT-PCR and WB analysis of cyclin D3 mRNA and protein expression level in ZNF652 overexpressed or downregulated A549 and H460 cells. **E** ZNF652 binding motif was predicted by the JASPAR database (http://jaspar.genereg.net/). **F** The potential binding sites of ZNF652 on the CCND3 promoter region were predicted by JASPAR. **G** Wildtype or mutant ZNF652 target sequence in CCND3 promoter. **H** The enrichment of ZNF652 on the CCND3 promoter was assessed via ChIP-PCR assay in A549 cells. **I** Luciferase reporter assay of CCND3 promoter activity in vector control and ZNF652-OE A549 cells. **J–L** Vector control cells and ZNF652-OE A549 and H460 cells were transfected with CCND3 plasmid. Cell viability was measured at 48 h (**J**). Cell cycle distribution was measured using flow cytometry analysis (**K**). Protein expression of cyclin D3 and CDK4 was examined using WB analysis (**L**). The data are presented as mean ± SD (*n* = 3). *p < 0.05, **p < 0.01, *vs*. Vector group; #*p* < 0.05, ##*p* < 0.01 *vs*. ZNF652-OE group.

ZNF652 belongs to the zinc finger protein family, which transcriptionally activates or represses its target genes [27, 28]. Cyclin D3 acts as a pivotal regulator of tumor proliferation and migration by regulating G1/S transition [29]. It has been reported that the deregulation of cyclin D3 is associated with unfavorable prognosis in NSCLC [17]. In the screening phase, we first analyzed the correlation between ZNF652 and CCND3 expression with 526 LUAD patient samples by ENCORI databases. It found that these two proteins were negatively correlated (r = −0.129, *p* = 0.003) (Fig. 8A). Cancertool analysis [30] showed an inverse relationship between ZNF652 and CCND3 (r = −0.1023, *p* = 0.0316) (Fig. S3). Additionally, based on TCGA data of LUAD (*n* = 518), ZNF652 was negatively correlated with CCND3 (Fig. S4). Next, we detected cyclin D3 expression in human LC tissues and normal adjacent tissues. WB analysis showed that the protein level of cyclin D3 was significantly higher in LC tissues compared with normal adjacent tissues (Fig. 8B). Thus, we speculated that ZNF652 might arrest the cell cycle in the G1 phase via transcriptionally repressing cyclin D3, leading to the abnormal growth of LC cells. To confirm this, we first determined the transcriptional repression of cyclin D3 by ZNF652 in LC cells. As shown in Fig. 8C, D, ectopic expression of ZNF652 dramatically inhibited the expression of cyclin D3 mRNA and protein levels, while ZNF652 knockdown significantly upregulated cyclin D3 mRNA and protein expression. These results indicated that ZNF652 negatively regulates cyclin D3 expression in LC cells.

Next, we predicted the binding motif of ZNF652 through the JASPAR database, which showed that ZNF652 has two potential binding sites in the 2000 bp promoter region before the transcription start site of CCND3 (Fig. 8E, F). ChIP-RCR experiments indicated that ZNF652 was enriched at both two sites in the CCND3 promoter regions (Fig. 8H). These results suggest that ZNF652 could directly bind to the promoter regions of CCND3 in LC cells. Furthermore, to verify that ZNF652 drives the transcription of CCND3, the CCND3 promoter (the 2000 bp promoter region before the transcription start site of CCND3) was cloned to the psiCHECK 2 luciferase vector. Meanwhile, the constructs of CCND3 promoter with mutant binding sites 1 and 2 on the ZNF652-binding sites were made (Fig. 8G). Luciferase assays showed that ZNF652 overexpression enhanced the promoter activity of wild-type CCND3, while did not affect the CCND3 promoter activity with mutant binding sites 1 or 2 (Fig. 8I). These results demonstrated that ZNF652 transcriptionally regulated CCND3 expression.

Then, to further explicit the role of CCND3 in the tumor-suppressive effect of ZNF652 in LC cells, we overexpressed CCND3 in ZNF652-OE A549 and H460 cells. The results showed that the inhibitory effects of ZNF652 on LC cell viability were significantly attenuated upon CCND3 overexpression (Fig. 8J). Meanwhile, CCND3 upregulation counteracted ZNF652-induced cell cycle arrest and decreased expression of cyclin D3 and CDK4 in LC cells (Fig. 8K, L). Collectively, these findings revealed that ZNF652 arrested the cell cycle at the G1 phase via transcriptionally repressing CCND3 expression.

### ZNF652 overexpression inhibited tumor growth in vivo

**Fig. 9:**
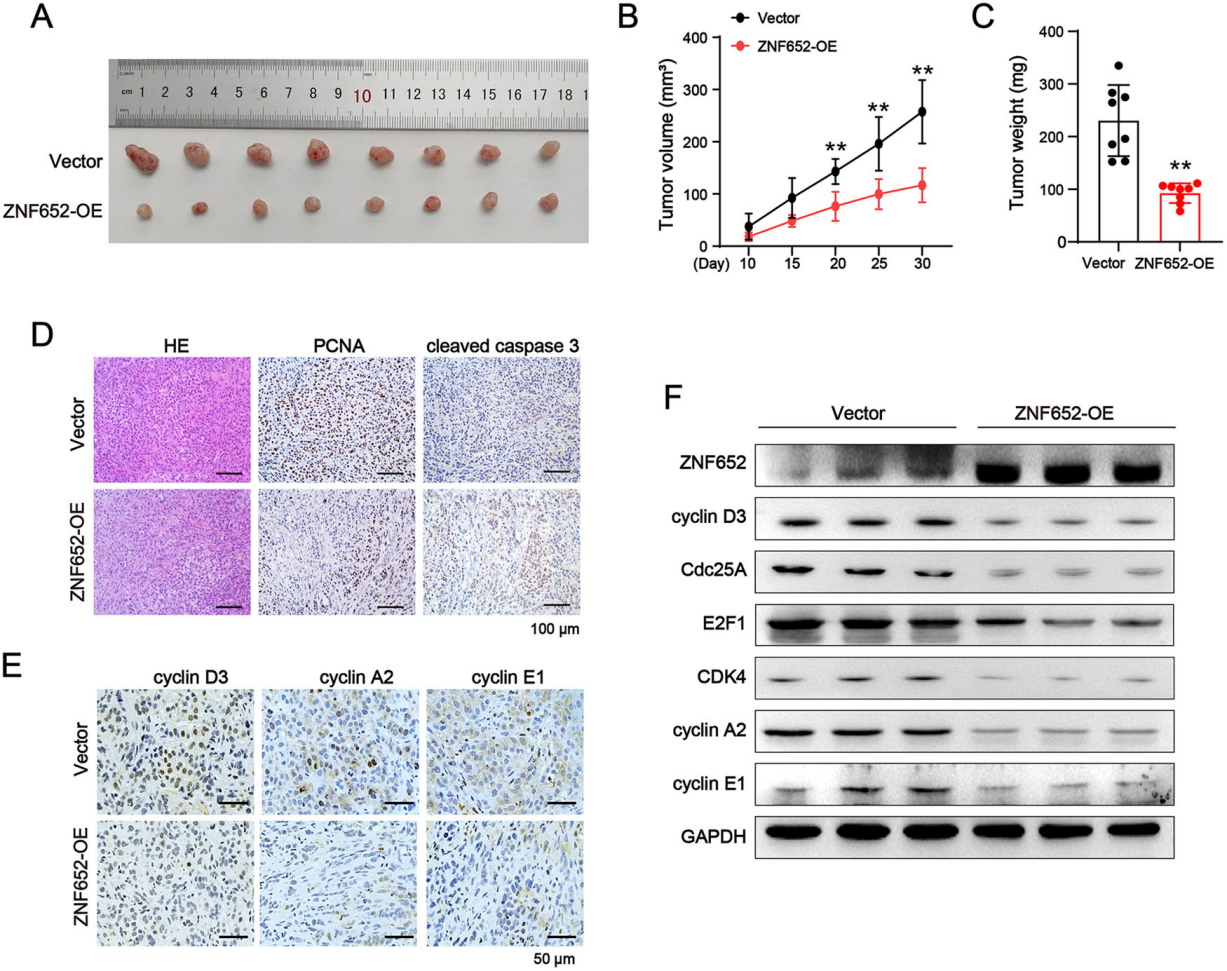
ZNF652 overexpression suppressed tumorigenesis of LC cells in vivo. ZNF652-OE or vector control A549 cells were subcutaneously injected into BALB/c nude mice at 5 × 10^6^ cells/mouse (*n* = 8 mice per group). **A** Images of the xenograft tumors. **B**, **C** Tumor volume and tumor weight were measured. **D**, **E** H&E staining of tumors and IHC staining of PCNA, cleaved caspase 3, cyclin D3, cyclin A2, and cyclin E1 expression. **F** WB analysis of ZNF652, cyclin D3, Cdc25A, E2F1, CDK4, cyclin A2, and cyclin E1 expression in tumors. The data are presented as mean ± SD (*n* = 8). **p < 0.01 vs. Vector group.

To further prove the tumor suppressive effect of ZNF652 on LC in vivo, ZNF652-OE or vector control A549 cells were subcutaneously transplanted into BALB/c nude mice. Growth curves of the tumors demonstrated reduced tumor progression in mice injected with ZNF652-OE LC cells, compared to mice that received vector control LC cells (Fig. 9A, B). Likewise, the tumor weight was lighter in the ZNF652 overexpression group than in the vector control group (Fig. 9C). Further, HE and IHC staining were conducted to assess tumor characteristics. Decreased PCNA and increased cleaved caspase 3 levels were found in ZNF652 overexpression tumor tissues (Fig. 9D). Additionally, the expression of cell cycle-related proteins cyclin D3, cyclin A2, and cyclin E1 were also reduced (Fig. 9E). WB analysis showed the expression of cell cycle-related proteins cyclin D3, Cdc25A, E2F1, CDK4, cyclin A2, and cyclin E1 were drastically reduced in ZNF652-OE groups, which was consistent with the in vitro results (Fig. 9F). These results show that ZNF652 overexpression inhibited the tumorigenesis of LC cells in vivo.

## Discussion

As a member of ZNFs, ZNF652 is the less studied and its biological function is still unknown. In this study, we revealed the tumor-suppressive role of ZNF652 in LC and explored its underlying mechanism. We found that ZNF652 was downregulated in LC tissues and cell lines. Function studies showed that overexpression of ZNF652 inhibited the malignant phenotype of LC in vitro and in vivo. Further investigation revealed that ZNF652 transcriptionally inhibited cyclin D3 expression, and thus arrested the cell cycle at the G1 phase. Findings from this study demonstrated that ZNF652 could be an important tumor suppressor in the development of LC.

Previous studies have reported that ZNF652 expression was reduced in many tumors and significantly correlated with tumor invasion [6, 31]. Recent research found that high expression of ZNF652 was positive with a good prognosis with LUAD [11]. Given that ZNF652 is located at chromosome band 17q, a region of frequent loss of heterozygosity in cancer, thus, we speculated that ZNF652 expression might be correlated to LC. Using data from different databases, online analysis tools, and experimental data, we observed a decreased expression of ZNF652 in LC tissues and cell lines. Moreover, reduced ZNF652 level is also associated with a higher tumor stage and shorter OS. We next explored the effects of ZNF652 on the malignant phenotype of LC cells. The LC cell lines with stable overexpression or knockdown of ZNF652 were established. We observed that ZNF652 overexpression not only significantly inhibited their proliferation, but also reversed the migration and invasion by regulating the expression of EMT-related markers (E-cadherin, ZO-1, Vimentin, and N-cadherin) in LC cells. Neilsen PM et al also reported that ZNF652 directly repressed key drivers of invasion and metastasis in breast cancer, including Vimentin, and TGFβ [25]. Furthermore, ZNF652 knockdown showed opposite effects in LC cell proliferation and metastasis. These results illustrated that ZNF652 might function as a tumor suppressor gene in LC cells.

Wondering the detailed mechanism of how ZNF652 inhibited LC proliferation and metastasis, we referenced RNA-sequencing results in ZNF652 overexpressed A549 cells. Analyses of gene expression profiles revealed that the DEG profile is particularly related to the cell cycle. Further study showed that these DEGs were mostly associated with G1-S phase transition. Taking these results together, we focused on the regulatory role of ZNF652 on the cell cycle. We performed flow cytometry, RT-PCR, and WB assays, which showed that ZNF652 overexpression blocked the cell cycle at the G1 phase and inhibited G1-S related gene expression, including p53, p21, cyclins, CDKs, E2F1, et al. Activation of p53 regulates various biological responses, including cell cycle arrest, apoptosis, and repair pathway [32], through inducing its transcriptional target p21 (CDKN1A), a Cdk inhibitor [33, 34]. Taking these results into consideration, we speculated that ZNF652 exhibited inhibitory effects on the proliferation and metastasis of LC cells by inducing cell cycle arrest.

Interestingly, in addition to the direct impact of ZNF652 on cell cycle, we found that ZNF652 overexpression led to cell senescence and enhanced the sensitivity of LC to CDDP. As an effective adjuvant treatment for LC, chemotherapy has improved the clinical outcomes of LC patients. Cisplatin, the first and most frequently used platinum-based drug, is a front-line chemotherapeutic agent for LC [35]. Notwithstanding, CDDP resistance limits its clinical utility and effectiveness in LC patients [36], which causes a complicated clinical problem. CDDP eradicates cancer cells by interfering with DNA synthesis and repair mechanisms, and subsequently activating apoptotic pathways [37]. In our study, we found that ZNF652 overexpression resulted in abnormal elevation of ROS production and DNA damage, ultimately leading to cellular senescence. Cellular senescence is a durable cell cycle arrest phenotype. Thus, ZNF652 overexpression-induced cell cycle arrest and cellular senescence inhibits LC progression, and also sensitizes LC cells to CDDP.

In previous research, ZNFs have been reported to work as transcriptional repressors or activators. For example, ZNF471 has been found to transcriptionally suppress the downstream targets by interacting with its corepressor [38]. ZNF638 physically interacted and transcriptionally cooperated with C/EBP to control PPARγ expression [39]. More importantly, it has been demonstrated that ZNF652 possesses transcription factor activity. For instance, Kumar R et al reported that ZNF652 participated in the tumorigenesis of breast and other tissues by forming a transcriptional corepressor complex [6]. Recent research illustrated that loss of ZNF652 upregulates PD-L1 transcription, and inhibits infiltrated CD8^+^ T cells in triple-negative breast cancer [10]. As such, we speculated that ZNF652 arrested the cell cycle at the G1 phase via transcriptionally regulation of its target genes.

The D-type cyclin family members are the important regulators of the G1-S transition [40]. In the present study, we found a negative correlation between ZNF652 expression and CCND3 level in LUAD. Moreover, the mRNA and protein expression of cyclin D3 was significantly reduced in ZNF652 overexpressed LC cells. Most importantly, we performed ChIP and dual-luciferase reporter assays, which showed that ZNF652 directly bound to the promoter of CCND3 and inhibited its transcription and expression, indicating a new regulation mechanism of CCND3. Besides, CCND3 upregulation restored the effects of ZNF652 on cell viability and G1-S phase transition, suggesting the function of cyclin D3 on the G1-S cell cycle in LC. In addition, Wang H et al reported that cyclin D3-CDK6 inhibition increased the cell amount of ROS, leading to subsequent cell apoptosis [41]. Combined with the previous study and our results, the elevation of ROS in ZNF652 overexpressed LC cells is associated with the inhibition of cyclin D3 by ZNF652. As such, we believed that ZNF652 could become a new target by which to influence the cell cycle, with benefit for LC patients. Our study provides novel insight into the molecular mechanism of action whereby ZNF652 acts as a tumor suppressor in LC.

## Conclusions

Collectively, we verified that ZNF652 inhibited the proliferation and metastasis of LC cells by directly binding to CCND3 and inhibiting the downstream cell cycle signaling. Thus, ZNF652 may present as a new tumor suppress gene in LC and as a potential novel target for the treatment of LC patients. Whether ZNF652 influences other biological processes is still unclear. Further study is needed to comprehensively analyze the function of ZNF652 in the lung or other kinds of cancer.

## Availability of data and materials

All data used during the current study available from the corresponding author on reasonable request.

## Supplementary information

Supplementary Figures and Tables

Original western blot
