# Supplementary material for: ZNF652 exerts a tumor suppressor role in lung cancer by transcriptionally downregulating cyclin D3
Source: Cell Death Dis. 2024 Nov 5;15(11):792. doi: 10.1038/s41419-024-07197-1 (PMC11538260; doi:10.1038/s41419-024-07197-1)
Supplement: Supplementary file 1 — Supplementary Figures and Tables [file 41419_2024_7197_MOESM1_ESM.docx]

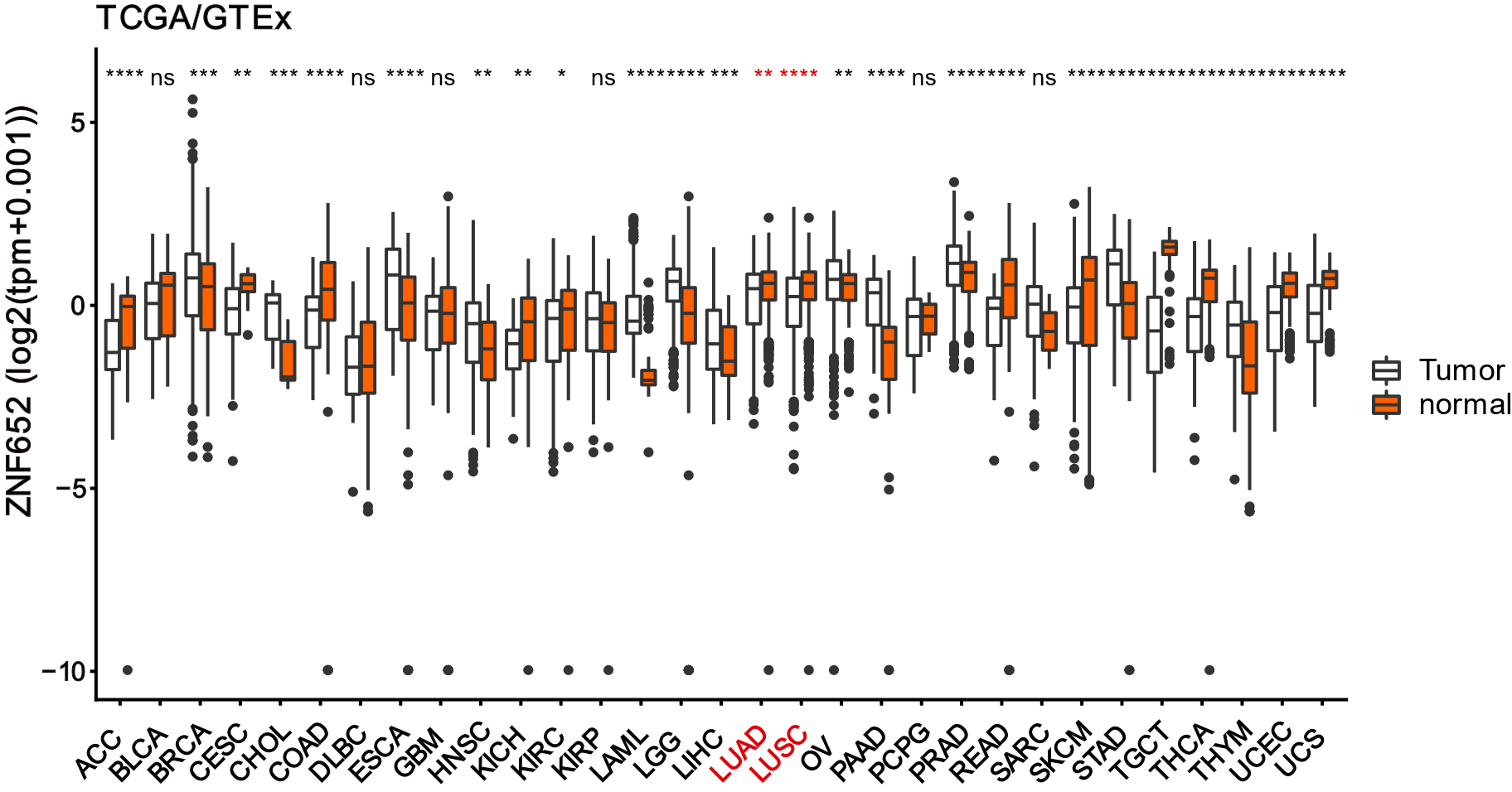


**Figure S1** ZNF652 transcript expression in TCGA/GTEx databases.


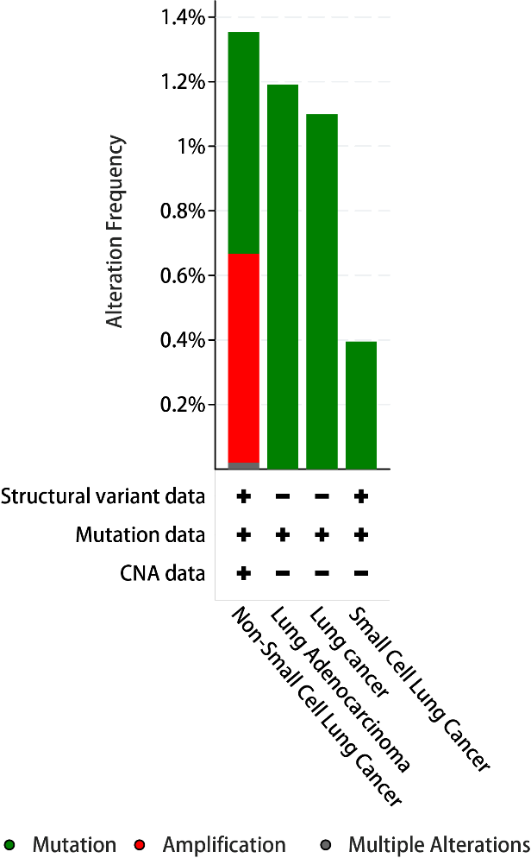


**Figure S2** The genome alternations of ZNF652 in lung cancer according to the cBioPortal database


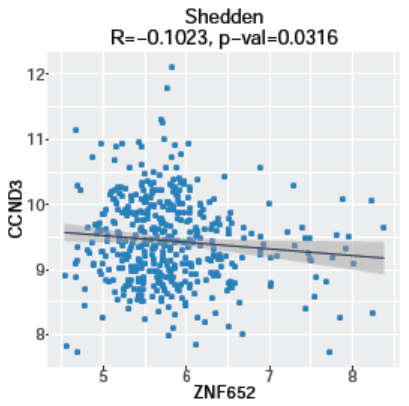


**Figure S3** The mRNA expression correlation between ZNF652 and CCND3 was analyzed using Cancertool.


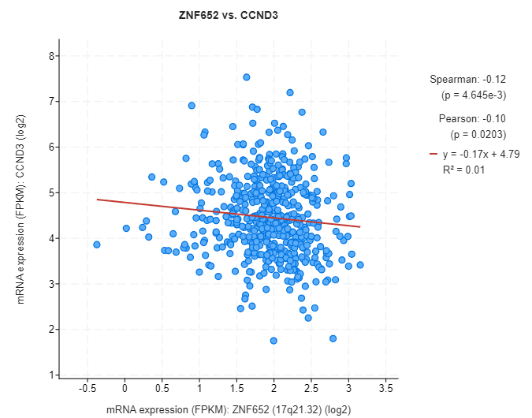


**Figure S4** The mRNA expression correlation between ZNF652 and CCND3 in LUAD from TCGA data was analyzed using cBioPortal database.

**Table S1.** Clinicopathological features of patients with NSCLC

| Clinicopathological features | Number of cases (n=30) |
| --- | --- |
| Age |  |
| ≤60 | 17 (56.7%) |
| ＞60 | 13 (43.3%) |
| Gender |  |
| Male | 25 (83.3%) |
| Female | 5 (16.7%) |
| Smoking Status |  |
| smoker | 19 (63.3%) |
| non-smoker | 11 (36.7%) |
| Differentiation |  |
| Low | 8 (26.7%) |
| Medium/High | 22 (73.3%) |
| TNM stage |  |
| Ⅰ | 11 (36.7%) |
| Ⅱ | 9 (30.0%) |
| Ⅲ | 10 (33.3%) |
| Histologic type |  |
| Adenocarcinoma | 16 (53.3%) |
| Squamous cell carcinoma | 14 (46.7%) |

**Table S2.** RT-PCR primer sequence

| **Gene** | **Forward primer (5’-3’)** | **Reverse primer (5’-3’)** |
| --- | --- | --- |
| ZNF652 | CTTCACCAGCAAACAGACTGTGAA | TTCTTTTCTGCATATCCATGGACG |
| E-cadherin | TCGACACCCGATTCAAAGTGG | TTCCAGAAACGGAGGCCTGAT |
| ZO-1 | GCAGCCACAACCAATTCATAG | GCAGACGATGTTCATAGTTTC |
| Vimentin | CCTTGACATTGAGATTGCCA | GTATCAACCAGAGGGAGTGA |
| N-cadherin | ATCAAGTGCCATTAGCCAAG | CTGAGCAGTGAATGTTGTCA |
| p53 | GGATGTTTGGGAGATGTAAGA | GCCAGCAGAGACTTGACAAC |
| p21 | GACACCACTGGAGGGTGACT | CAGGTCCACATGGTCTTCCT |
| cyclin D1 | CGTGGCCTCTAAGATGAAGG | TGCGGATGATCTGTTTGTTC |
| cyclin D3 | TACCCGCCATCCATGATCG | AGGCAGTCCACTTCAGTGC |
| GAPDH | CAAGGTCACCATGACAACTTTG | GTCCACCACCCTGTTGCTGTAG |
