# Supplementary material for: ZNF652 exerts a tumor suppressor role in lung cancer by transcriptionally downregulating cyclin D3
Source: Cell Death Dis. 2024 Nov 5;15(11):792. doi: 10.1038/s41419-024-07197-1 (PMC11538260; doi:10.1038/s41419-024-07197-1)

Figure 1D

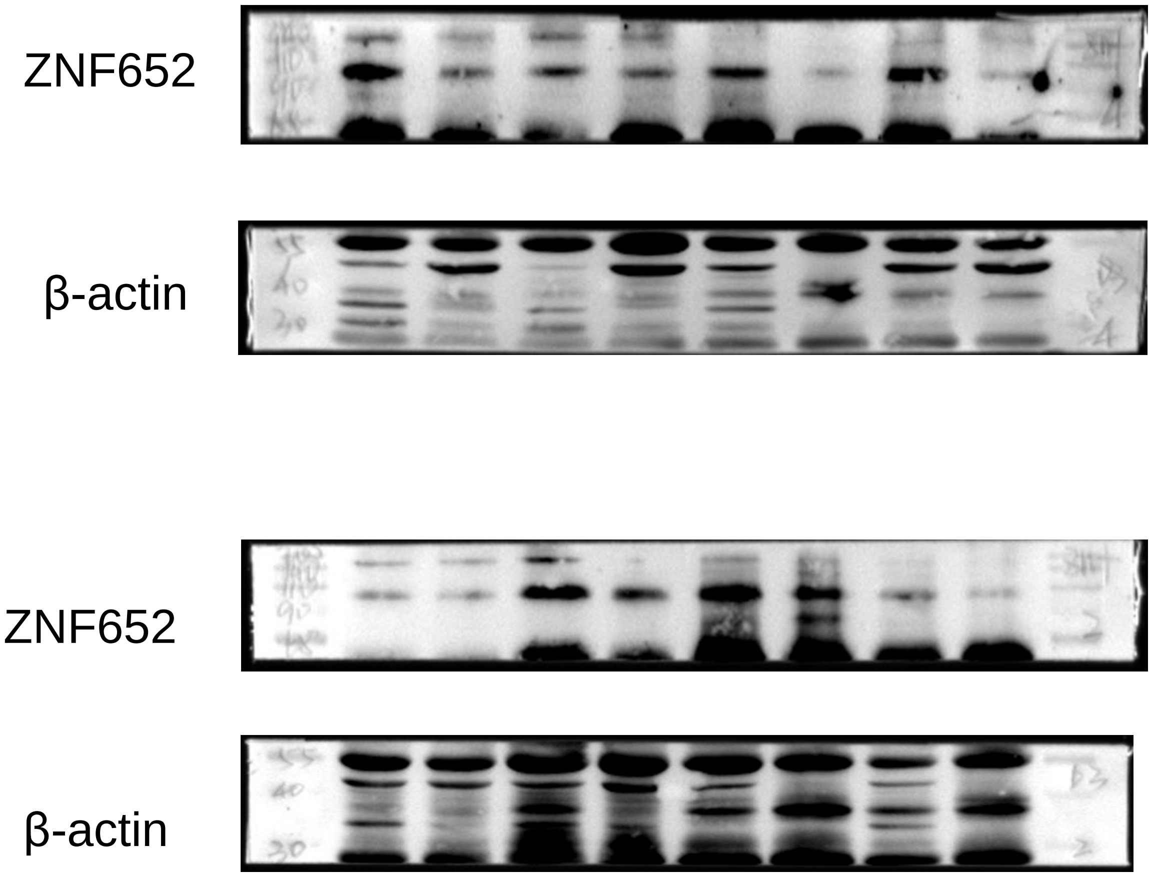

Figure 3A

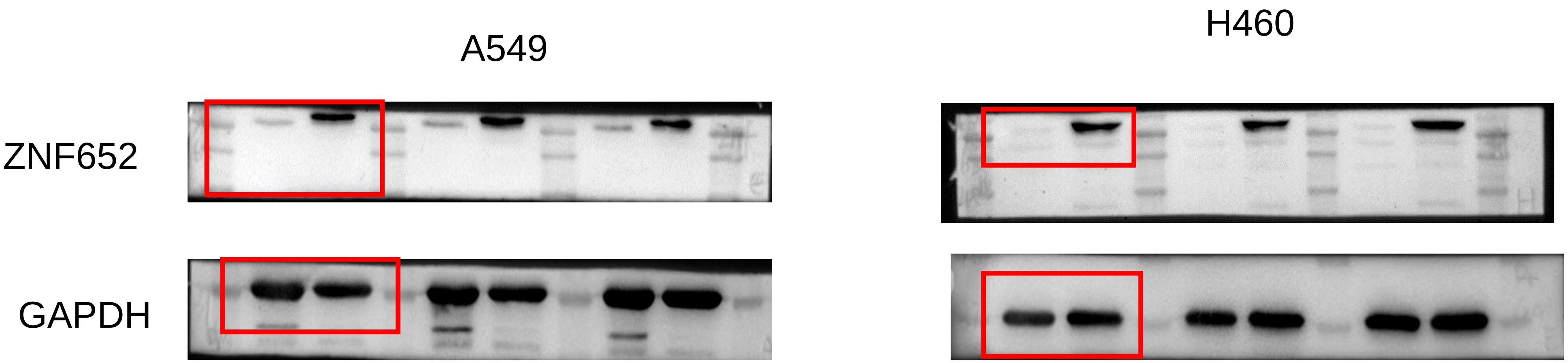

Figure 3E

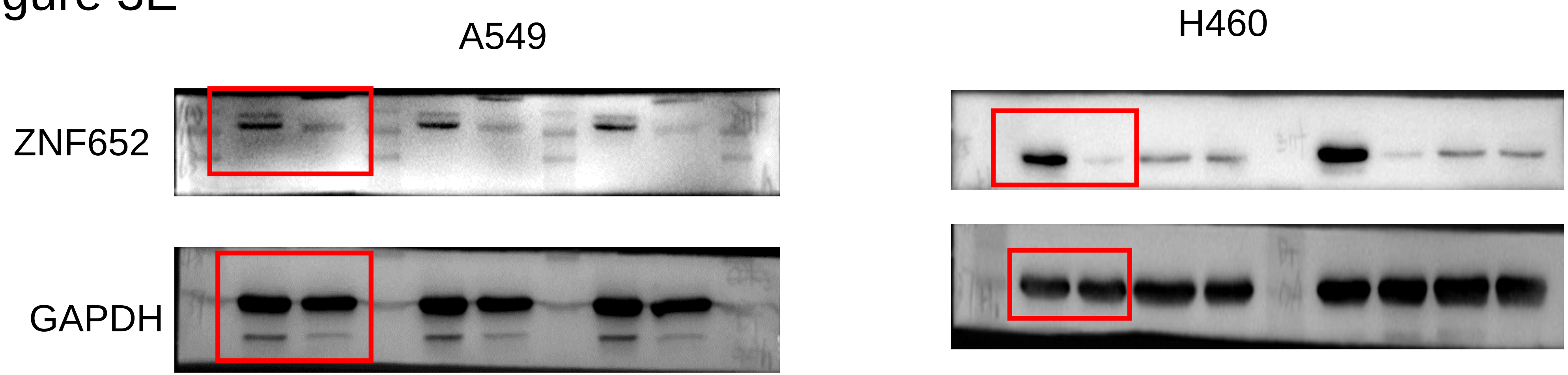

Figure 4G

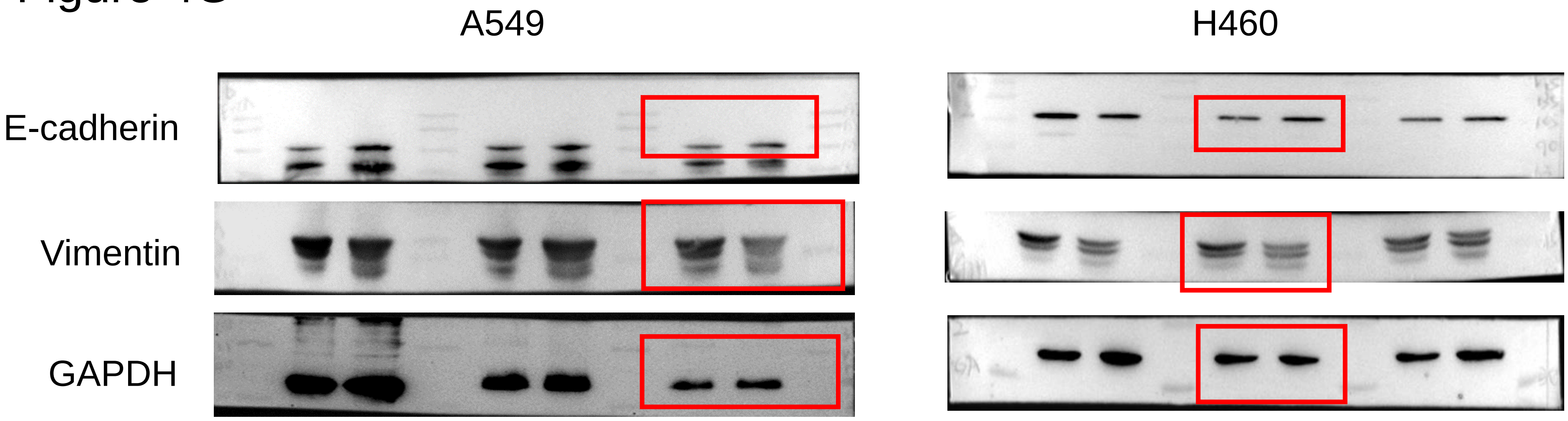

Figure 4H

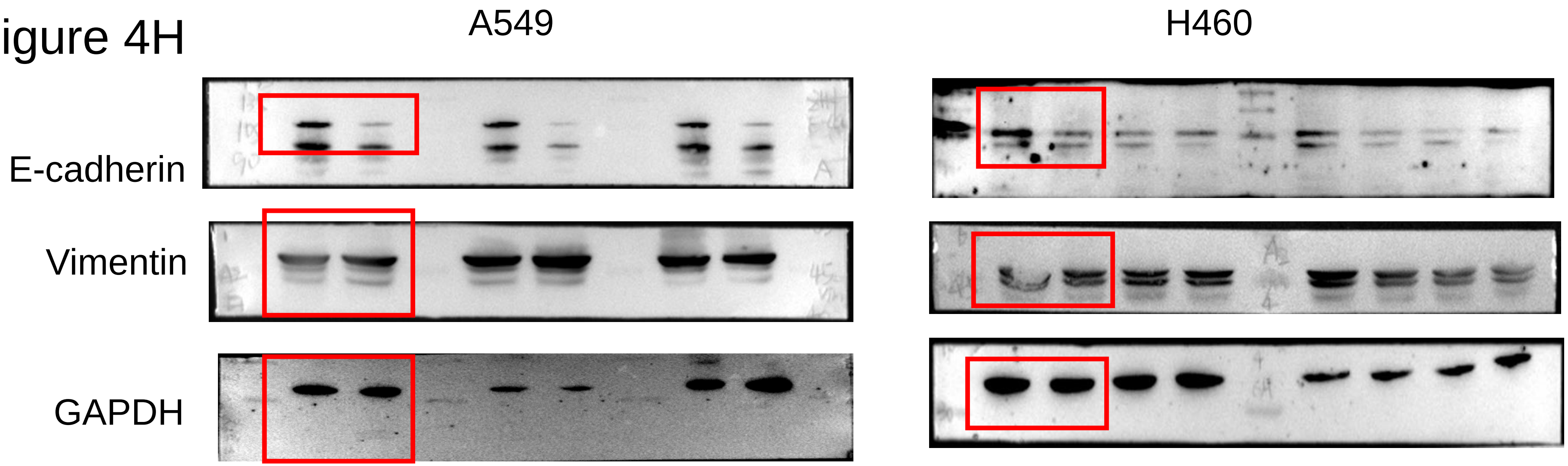

Figure 6C

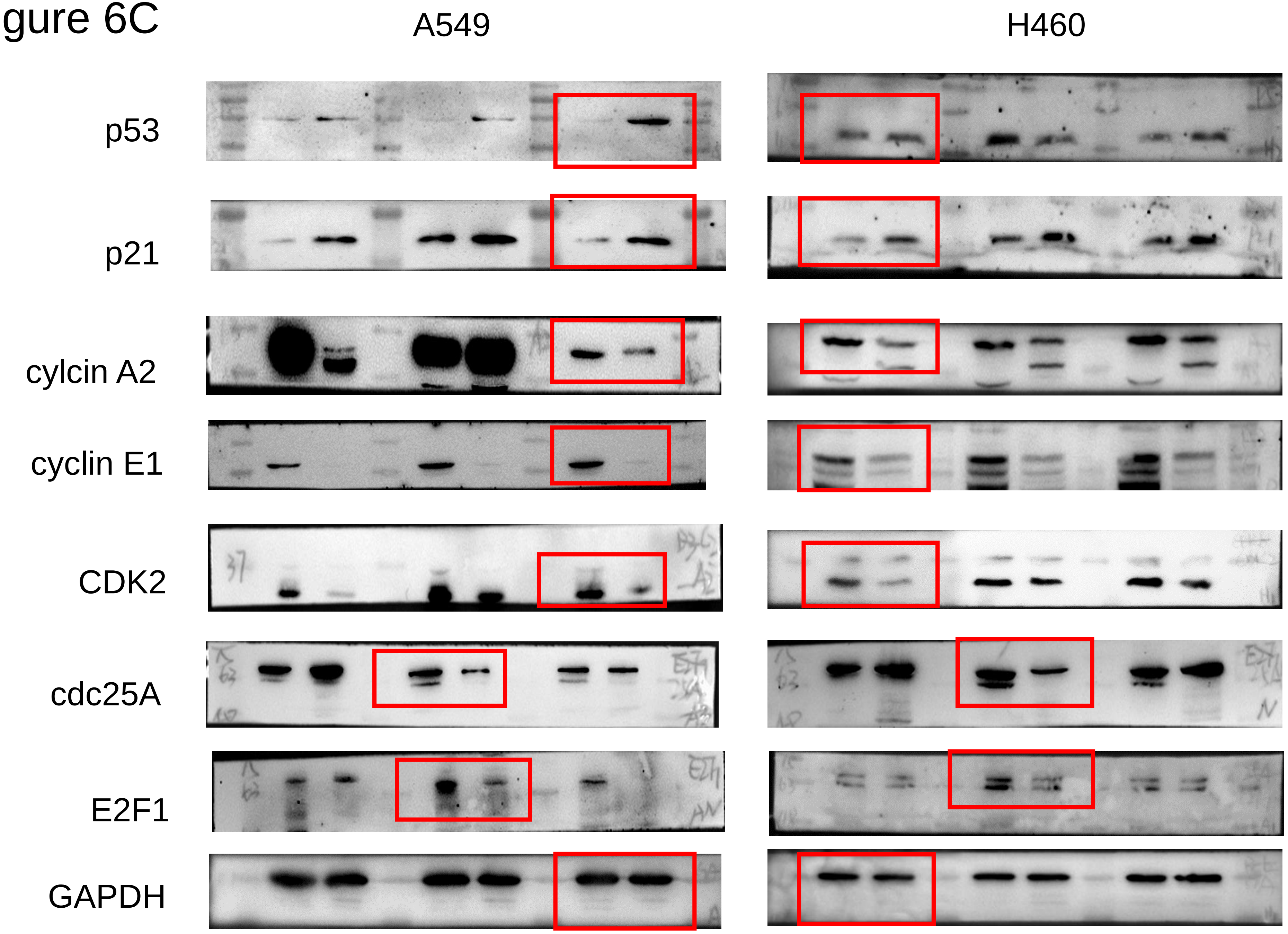

Figure 6D

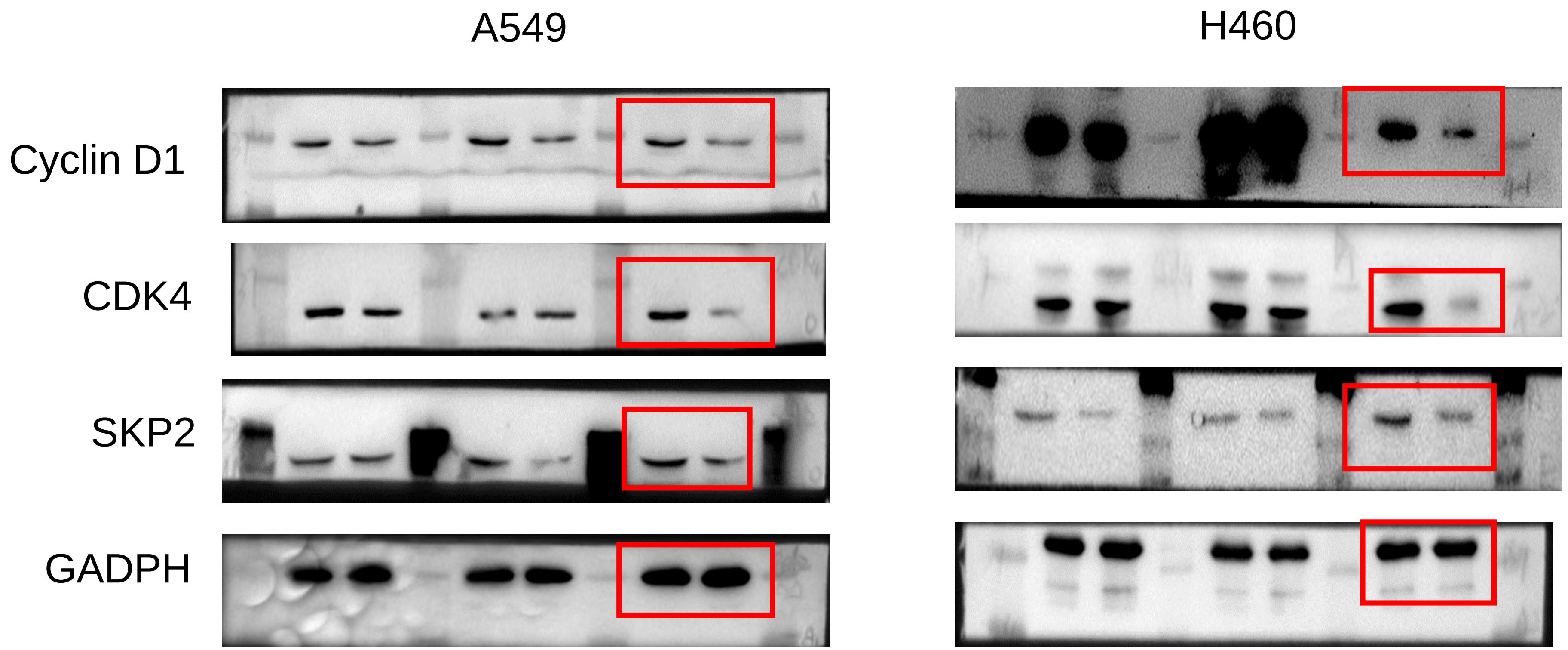

Figure 6G

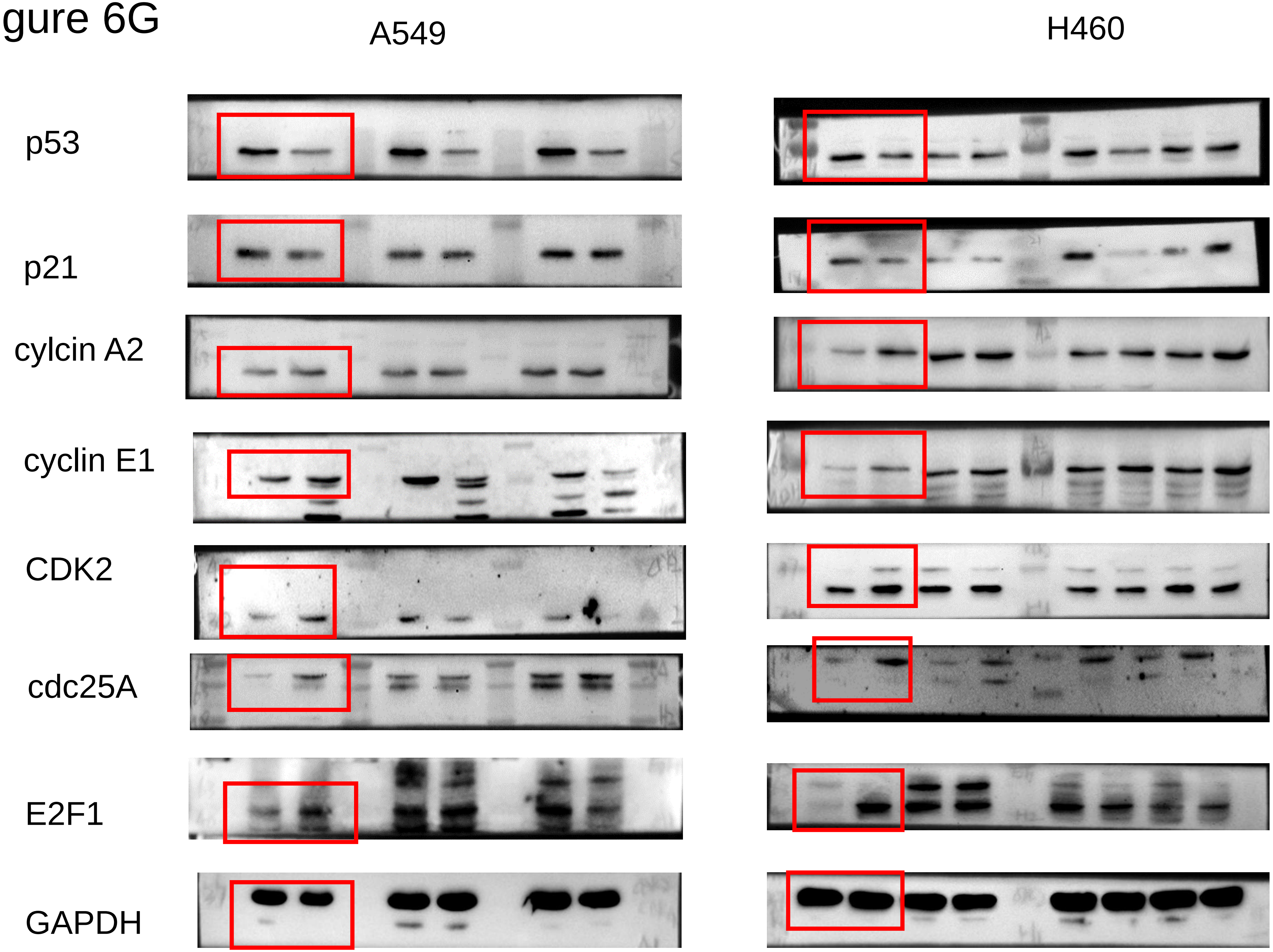

Figure 6H

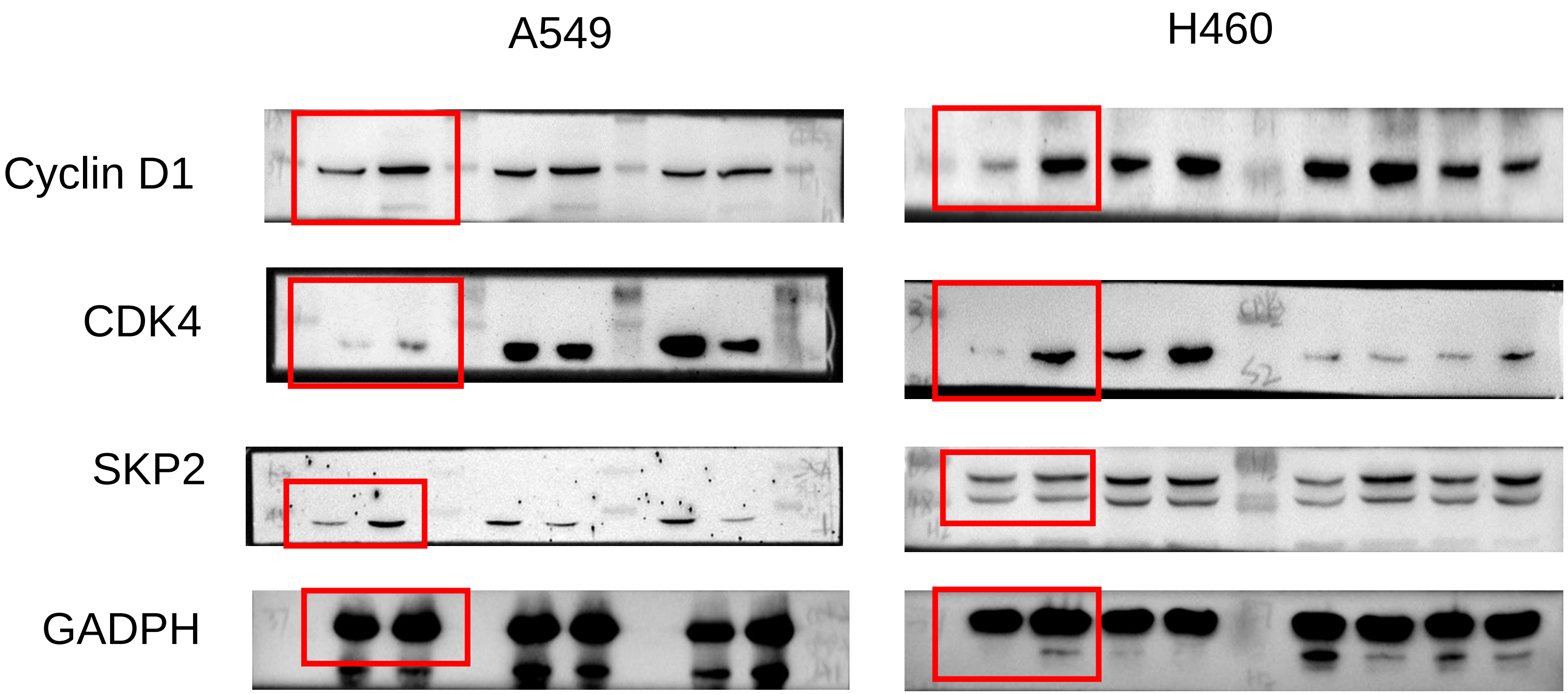

Figure 7B

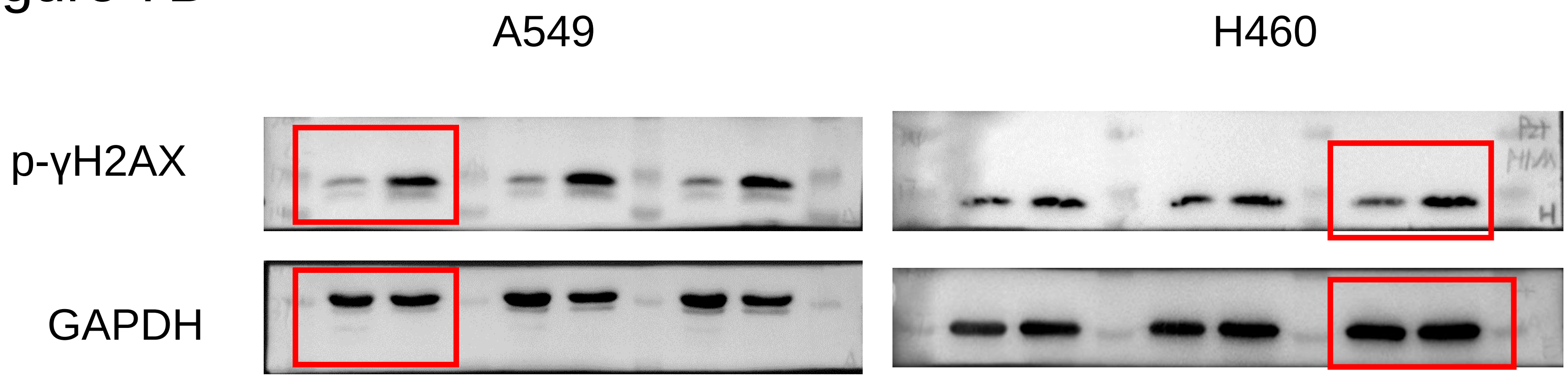

Figure 7F

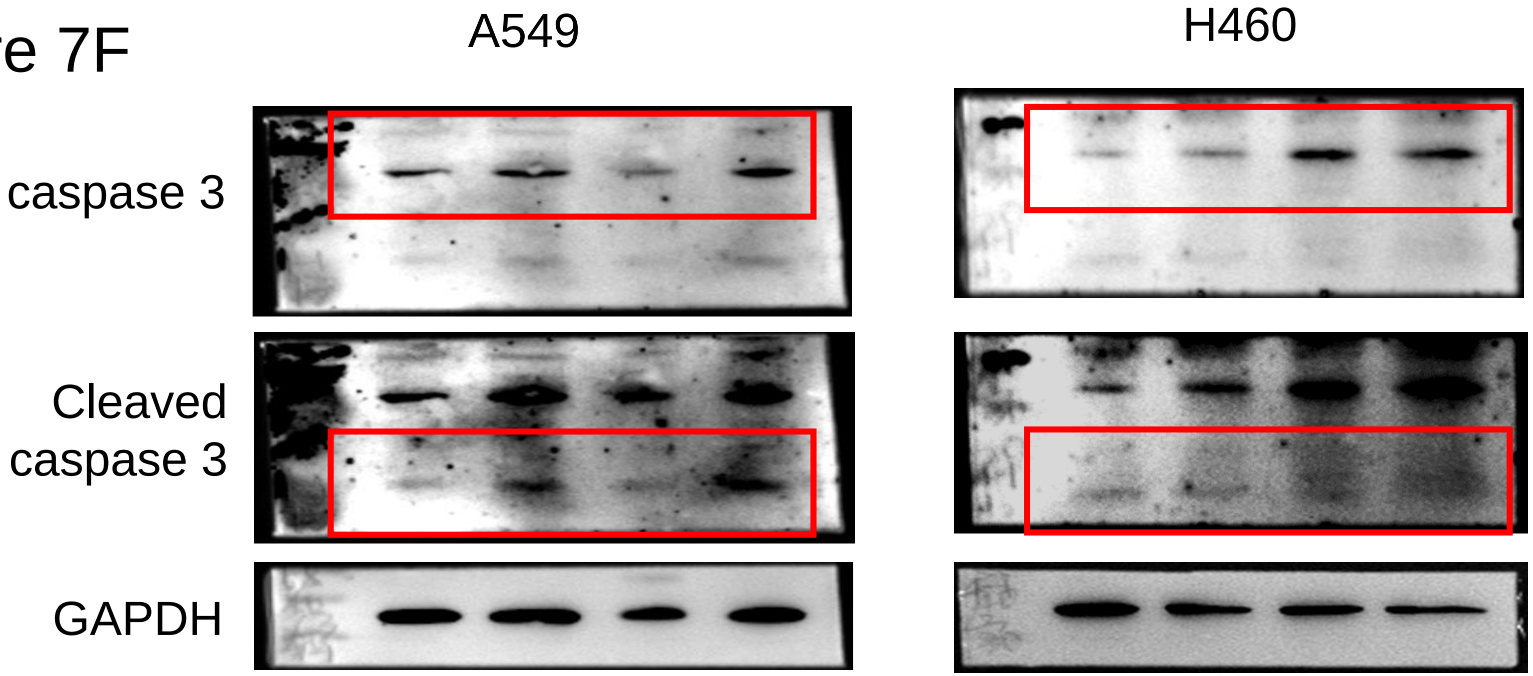

Figure 7H

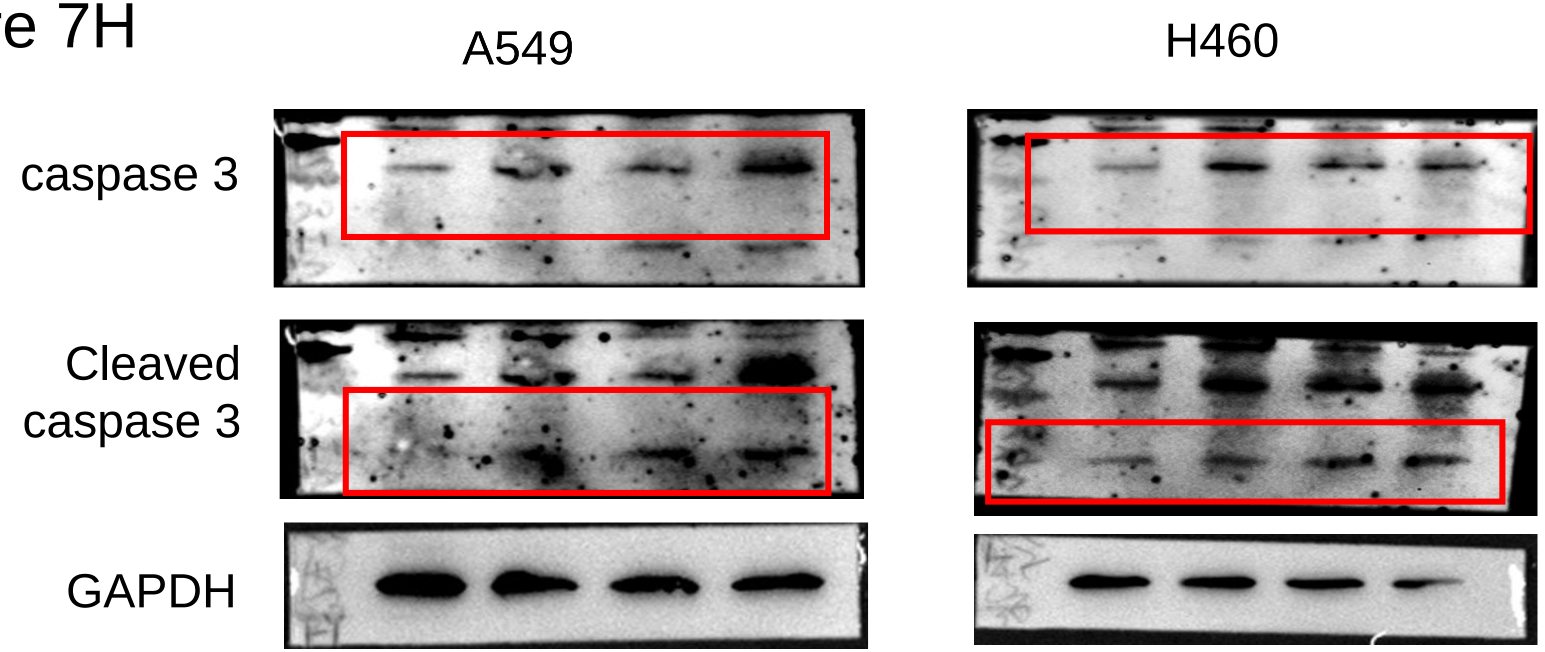

Figure 8B

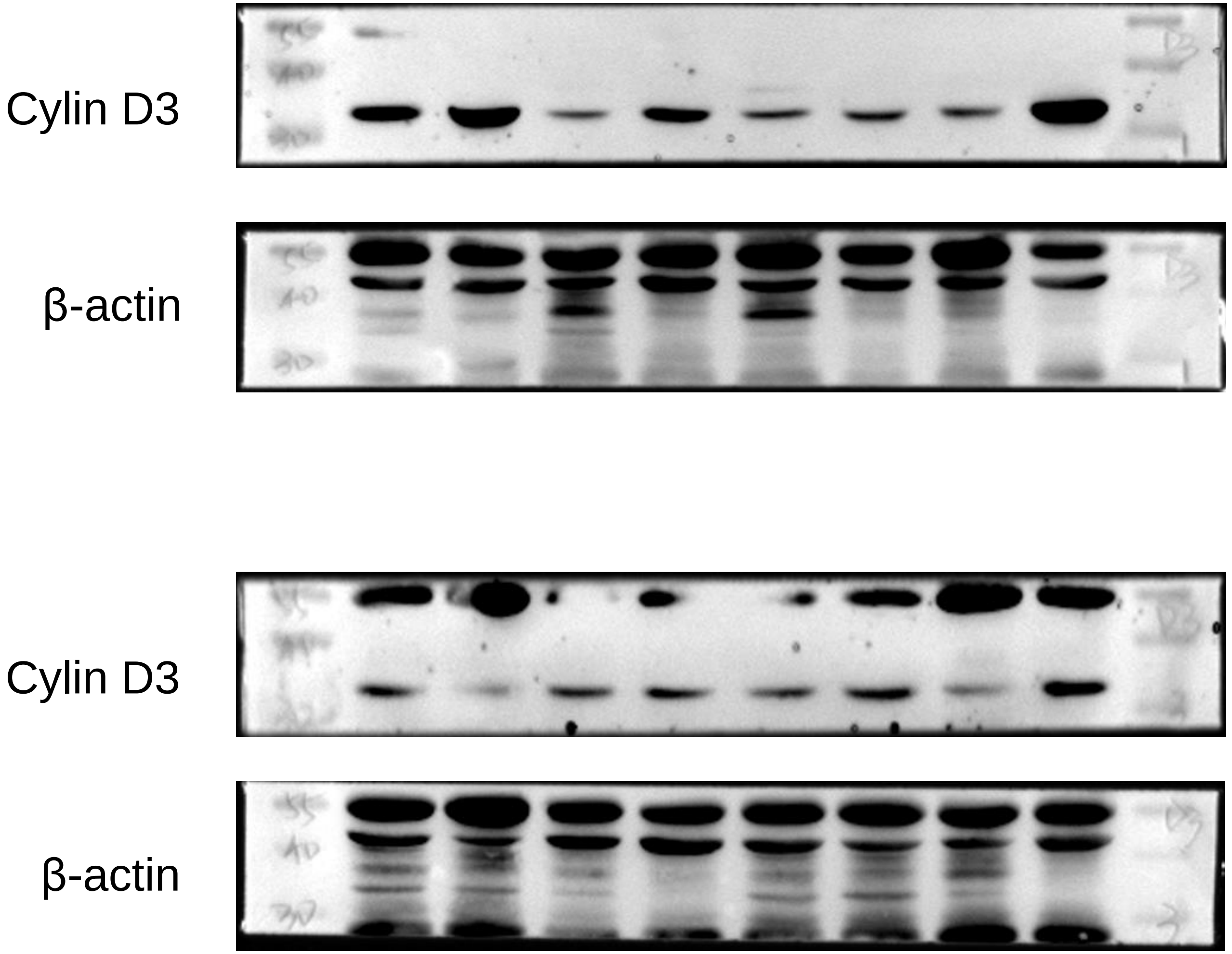

Figure 8C

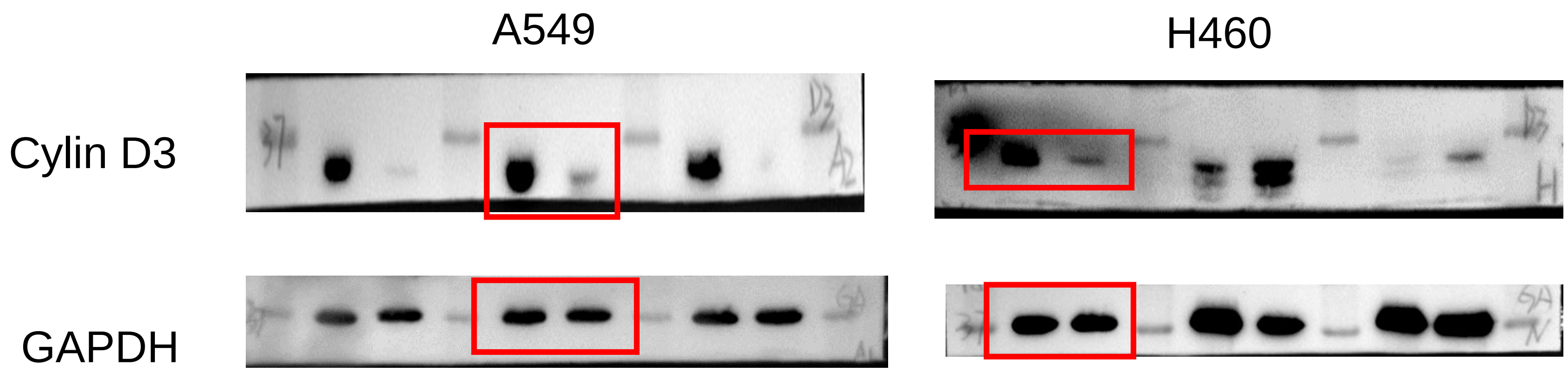

Figure 8D

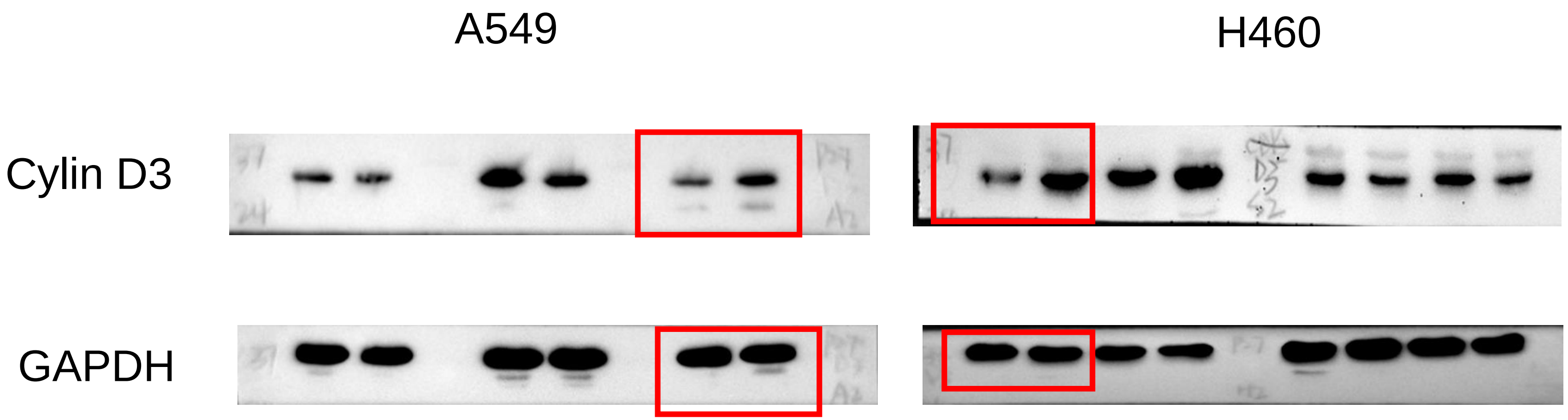

Figure 8L

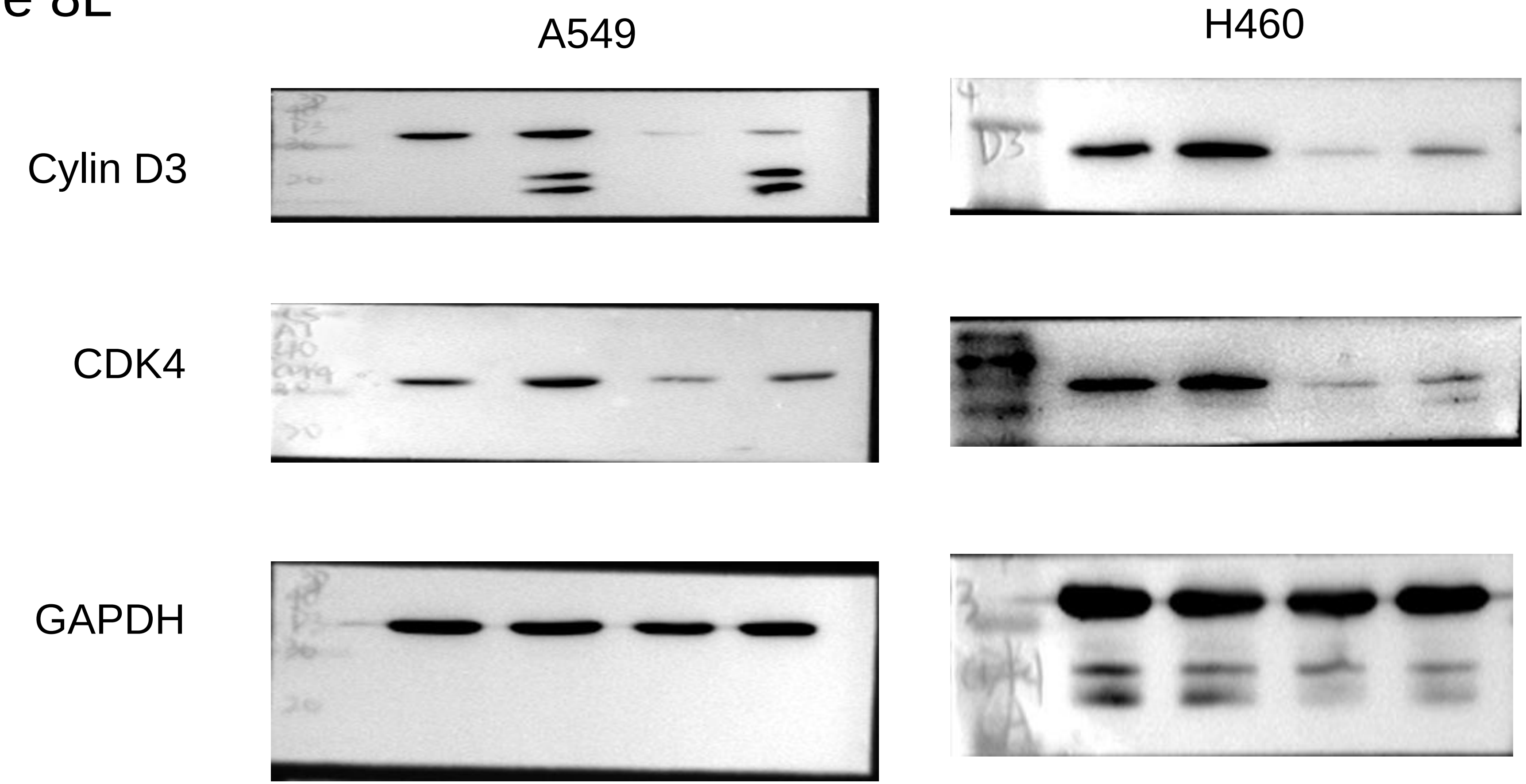

Figure 9F

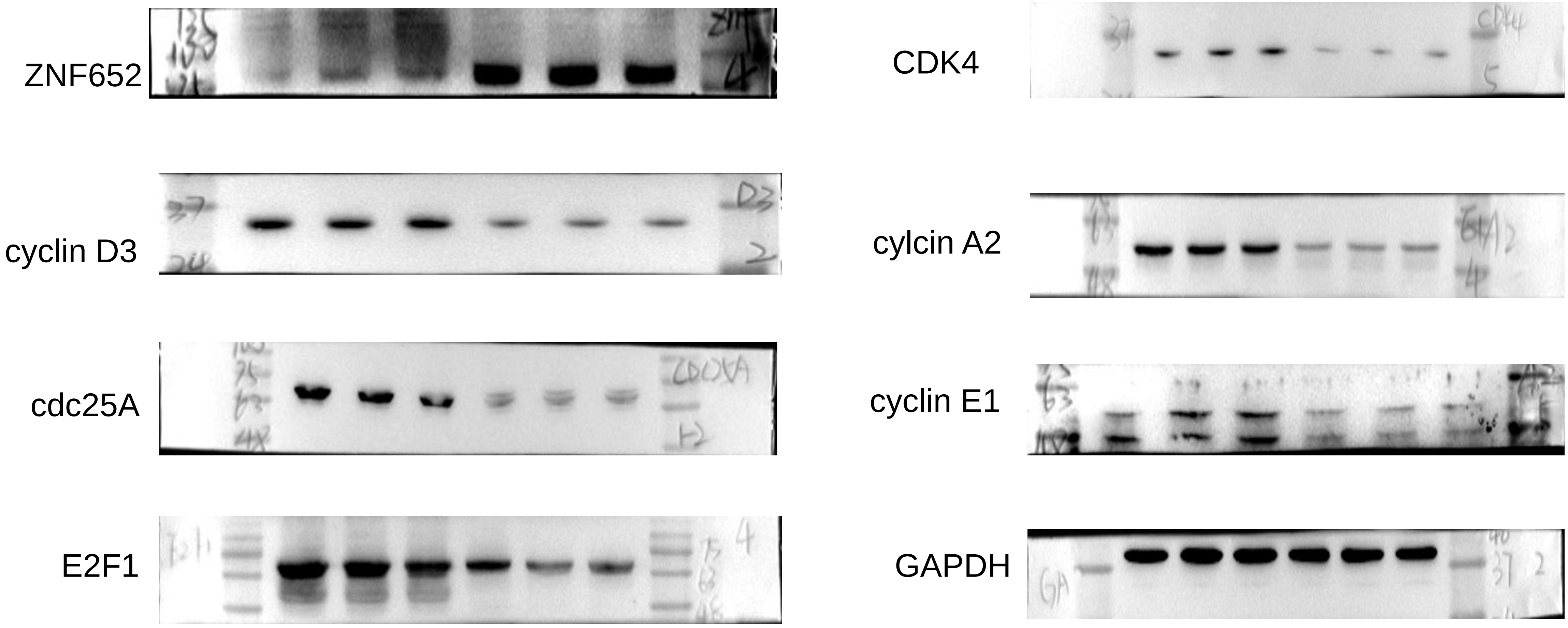

Supplement: Supplementary file 2 — Original western blot [file 41419_2024_7197_MOESM2_ESM.pdf]
